# Chlorinated biscoumarins inhibit chikungunya virus replication in cell-based and animal models

**DOI:** 10.1080/22221751.2025.2529889

**Published:** 2025-07-03

**Authors:** Chidinma Nelson Orji, Naphat Loeanurit, Van-Can Pham, Thi-Hong-Truc Phan, Kowit Hengphasatporn, Yasuteru Shigeta, Altri Diana Putri, Laura Sandra Lello, Andres Merits, Noppadol Wacharachaisurapol, Wanna Eiamart, Supeecha Wittayalertpanya, Tanatorn Khotavivattana, Warinthorn Chavasiri, Siwaporn Boonyasuppayakorn

**Affiliations:** a Center of Excellence in Applied Medical Virology, Department of Microbiology, Faculty of Medicine, Chulalongkorn University, Bangkok, Thailand; b Medical Science Program, Faculty of Medicine, Chulalongkorn University, Bangkok, Thailand; c Center of Excellence in Natural Products Chemistry, Department of Chemistry, Faculty of Science, Chulalongkorn University, Bangkok, Thailand; d Center for Computational Sciences, University of Tsukuba, Ibaraki, Japan; e Department of Biomedicine, School of Life Sciences, Indonesia International Institute for Life Sciences, Jakarta, Indonesia; f Institute of Bioengineering, University of Tartu, Tartu, Estonia; g Department of Pharmacology, Center of Excellence in Clinical Pharmacokinetics and Pharmacogenomics, Center of Excellence for Pediatric Infectious Diseases and Vaccines, Faculty of Medicine, Chulalongkorn University, Bangkok, Thailand; h Chula Pharmacokinetic Research Center, Faculty of Medicine, Chulalongkorn University, Bangkok, Thailand

**Keywords:** Biscoumarin, chikungunya virus, mice, efficacy, molecular docking, methyltransferase

## Abstract

Biscoumarin derivatives were evaluated for antiviral activity against chikungunya virus (CHIKV), a re-emerging mosquito-borne alphavirus with no approved treatment. Compounds 3 and 4 demonstrated potent *in vitro* antiviral efficacy, with EC₅₀ values of 2.85 ± 0.42 µM and 3.08 ± 0.45 µM (SI > 20) for compound 3 in Vero and HEK293 cells, respectively. Compound 4 showed comparable potency in Vero cells but was less effective in HEK293 cells. Time-of-addition and replicon assays suggested that both compounds act at a post-entry step, likely inhibiting viral RNA replication. *In vivo*, a single oral dose of 250 mg/kg was well tolerated in mice and rats, with no signs of acute hepatorenal toxicity and favourable pharmacokinetic profiles. Compound 3 & 4 significantly reduced tissue viral loads within 24 hours; however, their antiviral effect diminished after the drug was cleared from circulation. Due to concerns about potential cumulative toxicity, repeated administration was avoided. Preliminary mechanistic studies indicated moderate inhibition of the viral nsP1 methyltransferase and suggested possible involvement of host pathways. These findings highlight biscoumarin derivatives – particularly compound 3 – as promising antiviral candidates against CHIKV, meriting further optimization and investigation into their mechanisms of action.

## Introduction

Chikungunya virus (CHIKV) is a mosquito-borne alphavirus that has emerged as a significant global health concern, causing outbreaks in tropical and subtropical regions. It has affected over 110 countries in Asia, Africa, Europe, and the Americas [1]. CHIKV belongs to the family Togaviridae, genus alphavirus, and is primarily transmitted by *Aedes* mosquitoes. While the disease rarely leads to death, its hallmark symptoms of fever and severe, debilitating polyarthritis, often lasting for months, can greatly impact the quality of life [2]. CHIKV infection often leads to long-term morbidity, particularly in vulnerable populations. Outbreaks occur periodically every 5–10 years, and recent reports have documented co-circulation with flaviviruses such as dengue virus (DENV) and Zika virus (ZIKV). The co-infection cases were reported in Brazil and Thailand during ZIKV and DENV outbreaks and could potentially be related to severe clinical manifestations. Moreover, a recent 2019 outbreak showed that one-fifth of CHIKV-confirmed pediatric cases had severe manifestations, neurologic symptoms, or shock [3]. While no specific antiviral treatment exists, several vaccines are under development, with VLA1553 / IXCHIQ® recently approved for adults at risk of exposure [4,5]. Given the ongoing threat posed by CHIKV, exploring therapeutic options remains crucial.

In response to this medical need, extensive research efforts have been dedicated to developing antiviral agents targeting CHIKV. Various small molecules, including nucleoside analogs, viral protease inhibitors, and host-targeting agents, have been investigated for their potential efficacy against CHIKV. Among these, a promising candidate, favipiravir [6], progressed to preclinical and early clinical stages. However, no antiviral compounds have yet reached advanced clinical trials or received regulatory approval for CHIKV treatment. The continued pursuit of novel antiviral scaffolds with potent activity against CHIKV remains a critical focus in antiviral drug discovery.

Coumarins, also known as benzopyrones, comprise a fused benzene and α-pyrone ring system. They are widely distributed in nature, as more than 1,300 coumarin derivatives were found in plants, bacteria, and fungi. Coumarin is a potential drug candidate because of its stability, solubility, and low toxicity. Evidences have shown its inhibitory activity against many viral infections including those of HIV, influenza virus, enterovirus 71 (EV71) and coxsackievirus A16 (CVA16). At these cases, the targets included the virus-host interaction, anti-oxidative, and cellular response pathways. Biscoumarin, a subclass of the coumarin family, were recently highlighted for their potential biological activities. While best known for their anticoagulant properties [7], they also exhibited antimicrobials and anti-inflammatory effects. Three di-brominated derivatives (17-19) competitively inhibited lysosomal α-glucosidase (MAL12) of *Saccharomyces cerevisiae* with the IC_50_s of 0.62-3.31 µM [8]. Previous reports suggested that introducing a coumarin moiety as a drug conjugate can improve the ability to inhibit CHIKV infection [9,10]. Moreover, the derivatives were recently identified as inhibitors of methyltransferase of DENV and DENV replication (Loeanurit *et al*., under review).

Dengue virus (DENV) is another mosquito-borne flavivirus that poses a severe global health threat, often leading to hemorrhagic fever and shock syndrome. A series of halogenated biscoumarin anti-DENV screening identified two lead compounds, each bearing a single chlorine atom at the 3- or 4-position of the phenyl substituent on bis-4-hydroxycoumarin. These compounds exhibited strong anti-DENV efficacies across various cell lines, inhibited the activity of DENV2 NS5 methyltransferase, and significantly reduced viral translation and replication. Given their broad-spectrum activity against other dengue serotypes and Zika virus (ZIKV), these biscoumarin analogs represent promising antiviral leads. By extending this investigation to CHIKV, we aim to evaluate the potential of biscoumarins as a novel class of broad-spectrum antiviral agents targeting *Aedes* mosquito-borne arboviruses. In this work, we characterized two chlorinated biscoumarins, compound 3 (3,3′-((3-chlorophenyl)methylene)bis(4-hydroxy-2H-chromen-2-one)) and compound 4 (3,3′-((4-chlorophenyl)methylene)bis(4-hydroxy-2H-chromen-2-one)), for their anti-CHIKV efficacies in cell-based and animal models. The objective was to evaluate whether biscoumarins are suitable candidates for further development as drugs against mosquito-borne viruses.

## Materials and methods

### Compound synthesis

The compounds were synthesized and characterized for their chemical identities as previously described [8]. The compounds were dissolved in DMSO (PanReac AppliChem, Hesse, Germany) to the concentration of 50 mM, aliquoted, and stored at −20°C until use.

Sinefungin was purchased from Abcam, Cambridge, UK. Ribavirin was purchased from Bio Basic, Ontario, Canada. 5-iodotubercidin (5-IT) was purchased from MedChemExpress Monmouth Junction, NJ, USA.

### Cells and viruses

Vero (ATCC®CCL-81), LLC/MK2 (ATCC®CCL-7), C6/36 (ATCC®CRL-1660), and HEK 293 (ATCC®CRL-1573) cells were maintained as previously described [11-13]. CHIKV (ECSA genotype) was propagated in C6/36 cells as previously described [11,12].

### Efficacies and cytotoxicities of the lead compounds

Vero (5 × 10^4^) and HEK 293 cells (1 × 10^5^) were seeded in a 24-well plate and incubated at 37°C under 5% CO_2_ overnight. Cells were infected with CHIKV, and the selected compounds were analyzed for an effective concentration (EC_50_) using the plaque assay as previously described [14]. The compounds’ cytotoxicity (CC_50_) were assessed in parallel with the virus inhibition as previously described [15] Briefly, Vero (1 × 10^4^) and HEK 293 cells (1 × 10^4^) were seeded in a 96-well plate and incubated overnight. Various concentrations of the compound were added and incubated for two days. The cytotoxicities were analyzed by MTS assay. Results were reported as the mean and standard error of the mean (SEM) from three independent experiments. Selective index (SI) was the ratio of CC_50_/EC_50._

### Cell-based anti-attachment assay

Vero cells (5 × 10^4^) were seeded in a 24-well plate and incubated overnight. Cells were infected with CHIKV at MOI of 0.1 for 1 h at 37°C and treated with compounds at 10 µM at different stages – before, during or after virus uptake. (i) pre-treatment; cells were treated with the compound for 1 hour and then washed with PBS before exposure to virus, (ii) pre-incubation; virus was incubated with the compound for 1 h before infecting cells, (iii) co-incubation; virus and compound were added to cells simultaneously for 1 h, (iv) post-incubation; cells were infected with virus for 1 h and then washed with PBS before exposure to compound, and (v) throughout; compound present continuously during and after virus infection in cells. 1% DMSO was used as a control. Cells were incubated for 24 h. Supernatants were collected and viral titres were determined by plaque titration assay. Results were reported as percent inhibition from triplicate wells.

### Cell-based time-of-addition and time-of-removal assay

The time-of-addition and time-of-removal assays were used to characterize the compounds’ ability to interfere with viral replication. Vero cells (5 × 10^4^) were seeded in a 24-well plate and incubated overnight before being infected with CHIKV at MOI of 1 for 1 h with gentle rocking every 15 minutes. The compound at 10 µM was added or removed at 0, 1, 3, 6, 9, 12, and 24 h post infection (hpi).1% dimethylsulfoxide (DMSO) was used as vehicle control. Supernatants were collected and viral titres were determined by plaque titration assay. Three independent experiments were performed to confirm the findings.

### Replicon inhibition assay

Non-cytotoxic CHIKV replicon expressing *Renilla* luciferase (Rluc) reporter has been previously described [16]. The replicon was electroporated into BHK-21 cells and stable CHIKV replicon cell lines were selected in the presence of 5 µg/ml puromycin (Bio Basic, Ontario, Canada). The replicon cells (1 × 10^4^) were seeded in a 96-well plate and incubated overnight. The medium was removed, and a maintenance medium containing various concentrations of compounds was added. Cells were incubated for 24 h and were harvested to determine the replicon inhibition using *Renilla* luciferase assay system according to the manufacturer’s protocol (Promega, Medison, WI, USA). Briefly, the medium was removed, and the cells were washed with phosphate buffer saline. The lysis buffer was added to the plate and the plate was shaken for 15 min. Then, the substrate was added before measuring the luminescence using a VICTORTM X3 2030 Multilabel Reader (Perkin Elmer, Waltham, MA, USA). The signal was plotted into a non-linear regression curve fit, and the effective concentration 50 (EC_50_) was determined. Results were reported as mean and standard error of mean (SEM) from three independent experiments.

### In silico docking

The crystal structures of CHIKV envelope proteins (3N42 [17]), CHIKV nsP1 (8AOX [18,19]), CHIKV nsP2 (3TRK [20]), CHIKV nsP3 (3GPO [21]), and CHIKV nsP4 (7Y38 [22]) were used as protein receptors for ligand binding prediction. The missing residues of the nsP1 were added using Modeller [23] via Chimera UCSF version 1.17.1 [24]. We prepared the DFT-optimized structure of native inhibitors, including sinefungin (nsP1) [25], D160d (nsP2) [26], ADP-ribose (nsP3) [27], and Labmol-309 (nsP4) [28], as well as the 3D structure using gaussian16 with B3LYP/6-31G* basis set of theory [29]. A molecular docking of each compound with the viral targets was performed using AutoDock Vina 1.2.3 [30-32] to predict the preferential binding site of the compound for 10 iterated runs with random seed. Subsequently, the top pose from each run was chosen to compare with the binding energy of the native inhibitor of each viral target to identify the promising target for the compound.

### Methyltransferase assay

The nsP1 was expressed in a HEK293-TRex stable cell-line upon induction with 1 µg/ml doxycycline. Cells were grown in 150 ml DMEM in 500 mL flasks on a shaker at 180 rpm until they reached ∼1 × 10^6^ cells/mL before induction with doxycycline for 48 h. Cells were then collected and purified as previously described [33]. The methyltransferase activity was tested using MTase-Glo™ Methyltransferase Assay (Promega, Madison, WI, USA). The 2x reaction buffer included 100 mM Tris-HCl pH 7.5, 4 mM DTT, 20 mM KCl, 4 mM MgCl_2_, 2 mM GTP and 50 µM SAM. Compounds 3, 4, and sinefungin were added at 100 µM concentration to 10 µl of the 2x reaction buffer. Finally, nsP1 was added to the final concentration of 8.5 µM in the assay. The final volume of the reaction mix was 20 µl. DMSO was used as a mock treatment of compounds 3 and 4; whereas H_2_O was a mock treatment of the sinefungin. After incubating, 25 µL of MTase-Glo detection solution was added and reaction mixtures were incubated at room temperature for 60 min. The luminescence was measured as previously described. The percentage enzyme activity was calculated from two independent experiments.

### Animal experiment

#### Mice and rats

The Animal Care and Use Protocol was approved by the Institutional Animal Care and Use Committee of the Faculty of Medicine, Chulalongkorn University, Bangkok, Thailand, following the criteria of the National Institutes of Health, USA, for the use and treatment of laboratory animals (certificate number: 030/2566). The 8 – week – old C57BL/6 adult male mice and 7-week-old Sprague Dawley male rats were purchased from Nomura Siam International (Bangkok, Thailand).

#### Animal toxicity test

Eighteen C57BL/6 mice were equally divided into the compound treatment group and vehicle control group for toxicity study following a single dose (250 mg/kg) oral administration. The compounds were prepared in DMSO and added to the vehicle consisting of 35% polyethylene glycol 400, 2% ethanol and 63% deionized water before being orally administered to the treatment groups (n = 6). The control group (n = 6) was administered with 10% DMSO in the vehicle. The clinical signs were monitored daily and blood samples were taken on days 1, 3 and 7 post administration. Plasma samples were taken for analysis of alanine transaminase (ALT) (EnzyChrom, EALT-100, BioAssay, Hayward, CA, USA), and serum creatinine (Cr) (QuantiChromTM, DICT-500, BioAssay, Hayward, CA, USA). Survival curve was plotted. Additionally, to achieve pathological samples, one mouse was added to each group and intentionally sacrificed (censored) for liver and kidney pathological study by H & E staining.

Additionally, the dose was escalated to 500 mg/kg to observe the potential toxicity limit. Fifteen C57BL/6 mice were equally divided into the compound treatment groups (3 & 4) and vehicle control group. The compounds were prepared in DMSO and added to the vehicle consisting of 35% polyethylene glycol 400, 2% ethanol and 63% deionized water before being orally administered to the treatment groups (n = 5). The control group (n = 5) was administered with 10% DMSO in the vehicle. The clinical signs were monitored daily, and blood samples were taken on days 1, 3 and 7 post administration. Moreover, the compound 3 was analyzed in multiple doses manners. Briefly, the compound 3 (n = 5) was orally administered at 250 mg/kg, twice daily, for 3 consecutive days. The control group (n = 5) was administered with 10% DMSO in the vehicle under the same condition. Similarly, ribavirin (50 mg/kg) dissolved with PBS was administered twice daily for 3 consecutive days through the I.P route [34,35]. The clinical signs of compounds and vehicle treated groups were monitored daily, and blood samples were taken on day 4 post administration. Plasma samples were taken for analysis of alanine transaminase (ALT) (EnzyChrom, EALT-100, BioAssay, Hayward, CA, USA), and serum creatinine (Cr) (QuantiChromTM, DICT-500, BioAssay, Hayward, CA, USA). Survival curve was plotted. Additionally, to achieve pathological samples, one mouse was added to each group and intentionally sacrificed (censored) for liver and kidney pathological study by H & E staining.

#### Pharmacokinetic study

##### Sample preparation

To determine the levels of compounds 3 and 4 in mice and rat plasma, acetonitrile was used for sample extraction. Briefly, 1000 µl of acetonitrile was added to 20 µl or 10 µl of plasma containing compounds 3 or 4, respectively, mixed vigorously by vortexing, centrifuged, and the supernatant filtered through a nylon syringe filter (0.22 µm pore size) into a vial and placed in the autosampler at 15^o^C.

##### LC-MS/MS condition

Both compounds were analyzed using an LC-MS/MS: Shimadzu Corporation, Kyoto, Japan. The LC-MS/MS is comprised of Column Oven (CTO-20A), Detector (LCMS-8040), Autosampler (SIL-20ACXR), Pump (LC-20ADXR), Degasser unit (DGU-20A3R) and Valve unit (FCV-20AH2). Chromatographic conditions for the separation of the compounds include an Avantor ACE 3 µm C18 (100 × 3 mm) Column and a Phenomenex C18 (4.0 × 2.0 mm) Guard column. The column oven temperature was maintained at 40°C while the autosampler was maintained at 15°C. The mobile phase consisted of acetonitrile/0.1% formic acid (v/v) in water and a flow rate of 0.4 ml/minute with a total run time of 5 minutes. For quantification of the analytes, multiple reaction monitoring (MRM) in negative ion mode was used. Transitions of the product ions were at (m/z) 445.050→161.100 and 445.050→161.050 for compounds 3 and 4 respectively (Supplementary Figures S1.1–1.2)

##### 2.9.3.3. Validation of analytical method

The specificity of the method was done by comparing blank rat plasma with the spiked plasma of each compound to check for any interference in the retention time of both analytes. To evaluate the linearity of compound 4, eight concentration levels ranging from 0.098 to 50.000 µg/ml was used for the calibration curve while for compound 3, the eight concentration levels ranging from 0.0781 to 20.000 µg/ml was used for the calibration curve (Supplementary Figures S1.3 and 1.4, and supplementary Tables S1.1 and 1.2). Linear least-squares regression analysis (with the weighting factor of 1/C^2^ of the peak area of test standard versus the concentration) was used for plotting the calibration curve. The back-calculated concentrations for the analytes were between 89.770 between 111.224% for compound 4 and 93.853-105.021% for compound 3, which is within the accepted range of ±15%, except for the LLOQ where it was ±20% (Supplementary Figures S1.5-1.6). The coefficient of determination (R^2^) was greater than 0.99. The method was, therefore, valid and accurate for the quantification of both compounds.

##### 2.9.3.4. Pharmacokinetic experiment and data analysis

Six Sprague Dawley rats were divided into two treatment groups (n = 3 per group) of both compounds (3 and 4) for studying baseline pharmacokinetics. The compounds were orally administered at a dose of 250 mg/kg, and blood samples were collected through the tail vein at 15, 30 min, 1, 1.5, 2, 4, 8, 12, 24, 48, and 72 h after oral administration. In parallel, six C57BL/6 mice (n = 3 per group) were treated with a single high dose (250 mg/kg), and blood samples were collected at 6, 12, 24, 48, and 72 h after oral administration. All plasma samples were stored at −80°C until the analysis. The pharmacokinetic parameters were analyzed with PhoenixTM 64 version 8.3 (Certara, Princeton, NJ) using the non-compartmental model as previously described [14].

#### In vivo anti-CHIKV efficacy experiment

Ninety mice were divided into 6 groups – the mock infection group (n = 6), the virus infected-untreated group (n = 12), the infected vehicle-treated group (n = 18), the infected compound 3-treated group (n = 18), the infected compound 4-treated group (n = 18), and the drug control (ribavirin) group (n = 18). The mock infection group was injected with MEM, while the others were injected with CHIKV (5 × 10^7^ pfu/mouse) into their left footpad. After CHIKV infection, mice in the infected untreated group received no further treatment, while the 3-, 4-, and vehicle-treated groups received a single oral administration of 250 mg/kg of compounds or DMSO in the vehicle at 24 h after infection while the ribavirin-treated group received an I.P treatment of 50 mg/kg dose ribavirin twice daily. All mice were monitored daily for local inflammation (foot width and length), body temperature, and activity score. To measure the activity score, the scoring system as previously described was used [36,37]. Spontaneous physical activity/response and food intake of food were the parameters observed. The scores ranged from 1 (highest activity) to 5 (lowest activity). The rating of the scores are as follows – 1. very active, 2 – active 3. less active 4 – slow, 5 – lethargic. Blood and footpad tissue were sampled from each treatment group (n = 6/group/day) and the viral load by plaque titration assay. All samples were stored on ice immediately after collection and transferred to – 80 ° C within 15 min. The tissue samples were weighed and transferred to 2 ml tubes containing 2.38 mm metal beads (Qiagen, Hilden, Germany) and homogenized with the PowerLyzer 24 homogenizer (Qiagen, Hilden, Germany) following the manufacturer’s protocol. The samples were centrifuged at 4 ° C to clarify the supernatants. The virus titres in supernatants and serum samples were analyzed by plaque titration; viral RNA copy numbers were determined using RT-qPCR performed as previously described [14].

## Results

### Antiviral efficacy of chlorinated biscoumarins against CHIKV in mammalian cell lines

**Figure 1.**
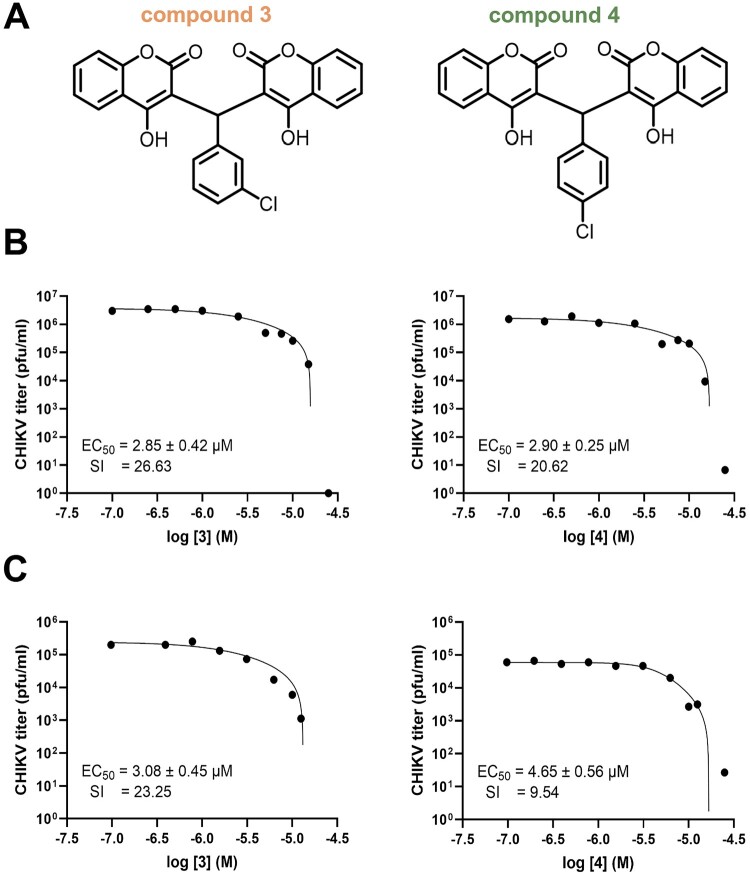
Antiviral activity of chlorinated biscoumarins against CHIKV in mammalian cells. (a) Chemical structures of compounds 3 and 4. (b) Dose – response curves showing CHIKV inhibition in Vero cells treated with compounds 3 and 4. Viral titres were measured by plaque assay at 24 hpi, and EC₅₀ values were calculated using non-linear regression. Selectivity indices (SI) were determined as the ratio of CC₅₀ to EC₅₀. (c) Dose-response curves showing CHIKV inhibition in HEK-293 cells treated with compounds 3 and 4 under identical conditions. Data are presented as mean ± SEM from three independent experiments performed in triplicate.

Two chlorinated biscoumarins, compounds 3 and 4 (Figure 1(a)), were simultaneously identified as potential anti-flaviviral agents (Loeanurit *et al*., under review), were evaluated for their antiviral activity against Chikungunya virus (CHIKV). The efficacy (EC₅₀) and cytotoxicity (CC₅₀) of each compound were assessed in both Vero and HEK-293 cells using plaque reduction assays and cell viability assays, respectively. In Vero cells, compounds 3 and 4 demonstrated comparable anti-CHIKV activities, with EC₅₀ values of 2.85 ± 0.42 µM and 2.90 ± 0.25 µM, and corresponding selectivity indices (SI) of 26.63 and 20.62, respectively (Figure 1(b)). The CC₅₀ values were 75.85 ± 3.25 µM for compound 3 and 66.97 ± 4.86 µM for compound 4 (Supplementary Figure 2). In HEK-293 cells, compound 3 again showed superior antiviral potency with an EC₅₀ of 3.08 ± 0.45 µM (SI = 23.25), while compound 4 exhibited a reduced efficacy (EC₅₀ = 4.65 ± 0.56 µM; SI = 9.54) (Figure 1(c)). Cytotoxicity values in HEK-293 cells were 71.60 ± 3.67 µM and 44.35 ± 1.48 µM for compounds 3 and 4, respectively (Supplementary Figure S2.1). The observed difference in antiviral activity between the two compounds across cell lines may reflect cell-type-specific differences in CHIKV replication kinetics or compound uptake. Nevertheless, compound 3 consistently exhibited slightly better potency and selectivity across both cell types. As the differences were not pronounced, both compounds were advanced for further mechanistic studies to identify their potential antiviral targets in the CHIKV replication cycle.

**Figure 2.**
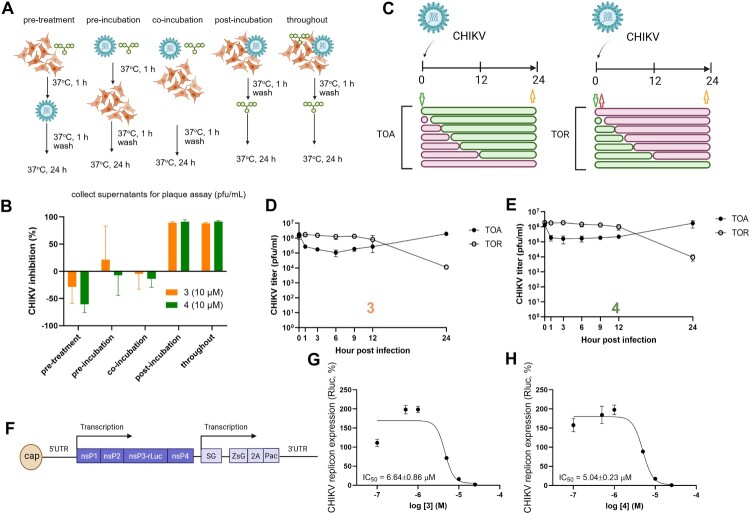
Compounds 3 and 4 inhibit CHIKV replication at a post-entry stage. (a) Schematic overview of the attachment inhibition assay. Vero cells were treated with compounds 3 or 4 (10 µM) during different phases of infection: pre-treatment (cells treated before virus exposure), pre-incubation (virus treated before cell exposure), co-incubation (virus and cells exposed to compound simultaneously), post-incubation (compound added after viral entry), and throughout (compound present continuously). (b) CHIKV inhibition (%) for each treatment condition was determined by plaque assay from culture supernatants at 24 h post-infection (hpi). Bars represent mean ± SD from triplicate wells. (c) Schematic of Time-of-Addition (TOA) and Time-of-Removal (TOR) assays. In TOA, compounds were added at various time points post-infection and maintained until 24 hpi. In TOR, compounds were added at 0 hpi and removed at designated time points. (d, e) CHIKV titres measured by plaque assay following TOA and TOR assays for compounds 3 (e) and 4 (e), showing a critical window of drug activity between 0–9 hpi. (f) Schematic of the CHIKV replicon construct encoding luciferase (Luc) and ZsGreen reporters, used to assess viral RNA replication. (g, h) Dose-response curves of compounds 3 (g) and 4 (h) in BHK-21 cells stably expressing the CHIKV replicon. Luciferase activity was measured at 24 h post-treatment to calculate EC₅₀ values. Data are shown as mean ± SD from three independent experiments.

To elucidate the stage of the CHIKV replication cycle targeted by compounds 3 and 4, we first conducted a series of attachment inhibition assays (Figure 2(a, b)). This assay was designed to determine whether the compounds interfered with viral attachment or entry into host cells. Neither compound exhibited significant inhibition when applied during the pre-treatment, pre-incubation, or co-incubation phases, suggesting a lack of virucidal activity or interference with virus-cell binding. In contrast, robust antiviral activity was observed when the compounds were added after viral entry (post-incubation) or maintained throughout the entire infection period, indicating a post-entry mechanism of action (Figure 2(b)). Noted that the compound pre-treatment enhanced the viral replication. A previous report suggested that a dicoumarol was a NQO1 inhibitor (NAD(P)H dehydrogenase, quinone 1) [38]. NQO1 is a key anti-HIV replication via the anti-oxidative stress pathway. Therefore, it could be possible that the compounds’ pre-treated cells were NQO1-impaired thus enhancing the viral replication.

To further define the timing of antiviral activity, we performed time-of-addition (TOA) and time-of-removal (TOR) assays (Figure 2(c–e)). In the TOA setup, compounds were added at various time points post-infection and maintained until 24 hours post-infection (hpi), allowing us to determine the latest time point at which the drug remained effective. A statistical analysis showed that the TOA of both compounds 3 and 4 were significantly different from their respective DMSO controls at early time-points (3–9 and 1–6 hpi, respectively) (supplementary Figure S2.2). Conversely, in the TOR assay, compounds were added at the time of infection and removed at defined intervals, identifying the earliest time point at which drug withdrawal led to a loss of antiviral efficacy. These complementary approaches pinpointed the critical window of drug activity within the early to mid-stages of CHIKV replication (0–9 hpi). Both compounds significantly reduced viral titres when added up to 9 hpi, with a modest reduction still observed at 12 hpi, suggesting that their primary antiviral effect occurs during early post-entry events, likely targeting viral genome replication or translation (Figure 2(d, e)).

To further confirm that compounds 3 and 4 act on CHIKV replication, we utilized a CHIKV replicon system (Figure 2(f)), in which a self-replicating RNA encoding a luciferase reporter was stably expressed in BHK-21 cells. Both compounds inhibited replicon activity in a dose-dependent manner, with calculated EC₅₀ values of 6.64 ± 0.86 μM and 5.04 ± 0.23 μM, respectively (Figure 2(g, h)). Cytotoxicity assays revealed CC₅₀ values of 100.55 ± 0.11 μM for compound 3 and 79.87 ± 0.40 μM for compound 4, confirming a favourable selectivity index.

Collectively, these findings demonstrate that the two chlorinated biscoumarins, compounds 3 and 4, act at a post-entry stage of CHIKV infection, likely interfering with viral RNA replication or early gene expression. These compounds represent promising antiviral leads warranting further mechanistic and structural optimization.

### Exploring CHIKV nsP1 MTase as one of the potential targets

**Figure 3.**
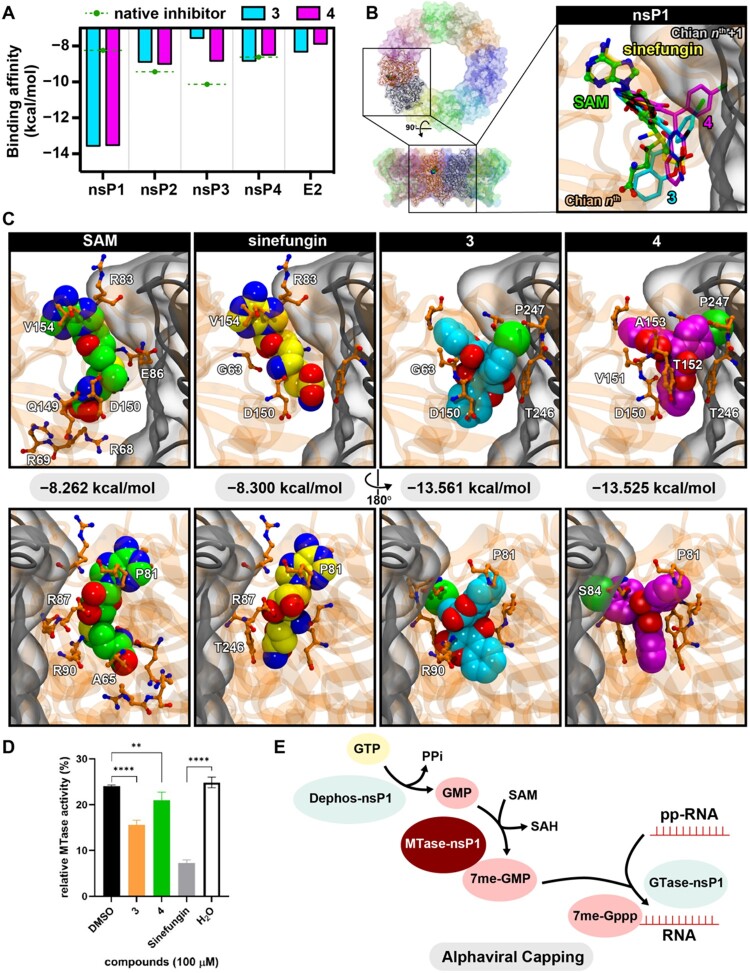
CHIKV methyltransferase binding and activities (a) in silico pan-docking of compounds 3 and 4 to viral proteins compared to their respective native inhibitors (green dash lines) (b, c) Binding poses, energy, and interacting residues of substrate (SAM), native inhibitor (Sinefungin), compounds 3 and 4 (d) *in vitro* methyltransferase assay (e) schematic diagram of alphavirus capping mechanism.

Previous studies identified compounds 3 and 4 as inhibitors of the dengue virus NS5 methyltransferase (MTase) by targeting the S-adenosyl-L-methionine (SAM) binding site (Loeanurit *et al*., under review), with similar IC₅₀ and EC₅₀ values across enzyme and cell-based assays. These compounds inhibited DENV RNA replication, as demonstrated by time-of-addition/removal experiments and DENV replicon assays. To determine whether CHIKV methyltransferase might also be a target, we conducted an *in silico* pan-docking analysis of compounds 3 and 4 against key CHIKV proteins, including the envelope protein (3N42), nsP1 (8AOX), nsP2 (3TRK), nsP3 (3GPO), and nsP4 (7Y38). The analysis identified CHIKV nsP1 methyltransferase as a promising candidate, with docking scores for both compounds approximately 1.6-fold higher than for the native SAM substrate (Figure 3(a)).

The CHIKV nsP1 MTase active site forms at a conserved pocket within its oligomeric structure (Figure 3(b)). Docking analyses revealed that SAM forms key hydrogen bonds with D87, E86, R69, and S84, as well as hydrophobic interactions with ALA65, VAL149, and PRO81. Sinefungin, a SAM analog and known MTase inhibitor, showed similar interactions, binding to E86, S84, R90, D150, and Y152. Compounds 3 and 4 bound to overlapping regions of the SAM-binding site, interacting with residues such as R90, E86, and D150. Additionally, compound 3 formed contacts with Y246 and Q149, while compound 4 interacted with Y152 and A153. These binding profiles suggest that compounds 3 and 4 are competitive ligands at the SAM-binding pocket, with comparable or superior docking scores relative to SAM and sinefungin.

To validate these predictions, we performed an *in vitro* methyltransferase inhibition assay using purified CHIKV nsP1. At 50 µM, compounds 3 and 4 significantly inhibited enzymatic activity compared to the DMSO-treated control (Figure 2(d)). However, their inhibition was not superior to that of sinefungin, which was dissolved in water, unlike compounds 3 and 4, which were solubilized in DMSO. This difference in solvent conditions may have influenced their relative activities *in vitro*. Despite this, the observed inhibition suggested that both compounds significantly interfered nsP1 MTase function, in which the compound 3 was more prominent than compound 4.

The CHIKV nsP1 enzyme is critical for 5′-capping of viral RNA, a process required for RNA stability and efficient translation in host cells (Figure 3(e)). Although our *in vitro* MTase results did not directly correlate with the docking results, it could be influenced by the solubility issue. By targeting this step, compounds 3 and 4 likely impair viral replication. In cell-based contexts, however, the capping process involves additional viral and host factors, which may contribute to the more potent antiviral effects observed in Vero and HEK-293 cells. Although our data supports nsP1 MTase as a plausible direct target, contributions from host-derived targets cannot be excluded. To address this possibility, we performed network-based target identification to explore additional cellular pathways potentially modulated by compounds 3 and 4 (Supplementary Figure S3 and Supplementary Table S3). Results showed that the PLK1, CASP6, and USP1 might be the potential targets. However, none of them has a direct involvement with CHIKV replication. Therefore, the potential host-derived targets remain elusive.

### Animal toxicity via oral administration

**Figure 4.**
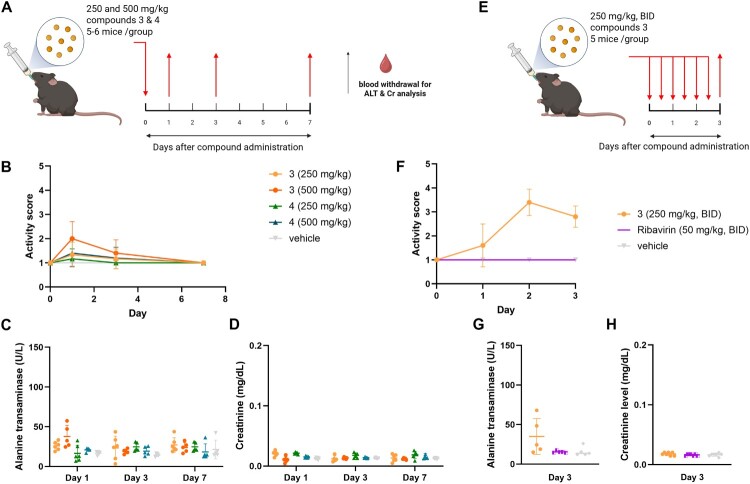
Assessment of acute oral toxicity of compounds 3 and 4 in mice. (a) Schematic representation of the single-dose toxicity study. C57BL/6 mice (5–6 per group) received a single oral dose of compounds 3 or 4 at 250 or 500 mg/kg. Blood samples were collected on days 1, 3, and 7 for liver (ALT) and kidney (creatinine) function tests. (b) Daily activity scores of mice monitored over a 7-day period following single-dose administration. (c, d) Serum levels of alanine transaminase (ALT, c) and creatinine (d) were measured on days 1, 3, and 7. (e) Schematic of the multiple-dose toxicity study. Compound 3 was administered orally at 250 mg/kg twice daily (BID) for 3 days. Ribavirin (50 mg/kg BID) and vehicle were used as controls. (f) Activity scores of mice during the multiple-dose study. (g, h) ALT (g) and creatinine (h) levels were measured on day 3 after the final dose.

To evaluate the safety of biscoumarin analogs, acute oral toxicity studies were conducted in C57BL/6 mice. Mice were given a single oral dose of compounds 3 or 4 at either 250 or 500 mg/kg. All animals survived through the 7-day observation period without visible clinical signs of toxicity (Supplementary Figure S4). Daily monitoring showed no significant difference in activity scores among groups (Figure 4(b)), and liver (ALT) and kidney (creatinine) biomarkers remained within normal limits (Figure 4(c, d)). Mild, transient hepatocellular vacuolization was observed in liver tissues at day 1, including in vehicle-treated animals, while kidney histology remained within normal limits (Supplementary Figure S5). Although serum ALT levels showed a slight increase on day 1 in the 500 mg/kg group of compound 3, this was not statistically significant and resolved over time, suggesting transient hepatic stress.

To assess tolerance under repeat dosing, compound 3 was administered at 250 mg/kg twice daily (BID) for three days. Ribavirin, a known antiviral agent, was included as a positive control at 50 mg/kg BID, intraperitoneally [34,35] In the compound 3 BID group, activity scores increased by day 2, indicating reduced overall physical activity (Figure 4(f)). In contrast, ribavirin-treated mice maintained normal behaviour and activity throughout. By day 3, serum ALT levels in the compound 3 BID group were markedly elevated compared to both vehicle and ribavirin groups (Figure 4(g)), while creatinine levels remained within normal limits across all groups (Figure 4(h)). Histopathology of liver tissues from compound 3-treated mice revealed hepatocellular vacuolization and mononuclear infiltration, suggesting acute liver injury and drug-induced hepatitis (Supplementary Figure S6). Renal tissues also showed signs of proximal tubular edema and vacuolization, consistent with mild renal toxicity. No pathological abnormalities were observed in ribavirin-treated animals.

These findings indicate that while a single oral dose of compound 3 or 4 at up to 500 mg/kg was well tolerated, repeated high dosing of compound 3 may lead to hepatotoxicity and renal tubular injury. In contrast, ribavirin was well tolerated under the same dosing schedule, reinforcing its utility as a reference for acceptable safety. Based on these results, 250 mg/kg as a single dose was selected as the highest safe concentration for further pharmacokinetic and efficacy studies.

### Pharmacokinetics in rodents

During the validation of the LC-MS/MS based method, both compounds were separated without any significant interference. The retention time of compounds 3 and 4 were 3.3 and 3.4 mins, respectively (supplementary Figures S1.5 and S1.6). The concentration of the calibration curve of 3 and 4 ranged 0.0781–20.000 and 0.098–50.000 µg/ml, respectively (supplementary Figures S1.3 and S1.4). The regression equations were Y = (17172.4)X + (−174.641) and Y = (9345.45)X + (−172.133) for compounds 3 and 4, respectively (supplementary Figures S1.3 and S1.4). The back-calculated concentrations for the analytes were between 89.770 and 111.224% and R^2^ were greater than 0.99 (supplementary Tables S1.1-1.2).

**Figure 5.**
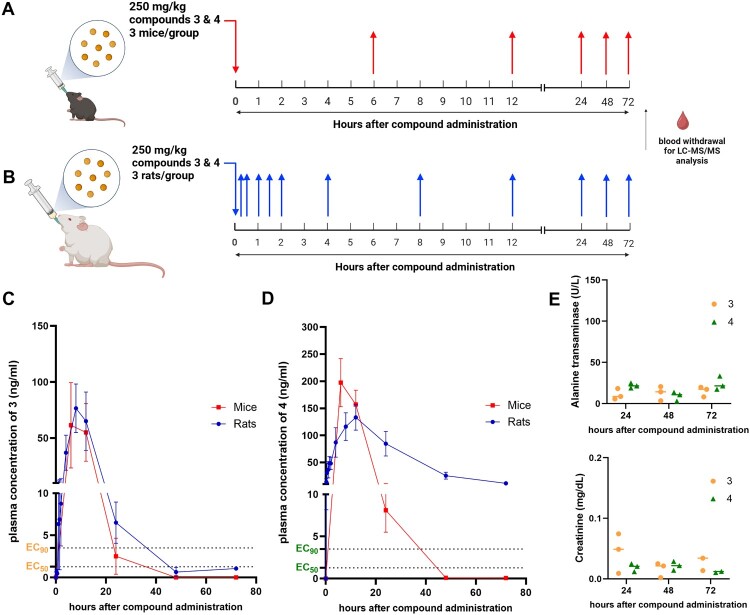
Plasma-concentration time profile of compounds 3 and 4 in mice and rats (a, b) A scheme of experiment in mice (a) and rats (b). Time points used for collection of plasma samples are indicated by arrows pointing up. (c, d) Results of plasma-concentration time profile of 3 and 4 were plotted in means and standard deviation (n = 3), comparing between mice and rats. The EC_50_ and EC_90_ levels of 3 and 4 are marked with dotted lines. (e) The ALT and Cr levels were evaluated in rats at 24, 48, and 72 h after compounds’ administration.

A single dose (250 mg/kg) of each compound was orally administered to groups of mice and rats (n = 3/group) (Figure 5(a, b)). Blood samples were taken at various time points (Figure 5(a, b)) for analysis of plasma concentration-time profiles (Table 1). The peak plasma concentrations and time (C_max_ and T_max_) of compound 3 were 68.9 ng/mL at 10 h in mice and 80.69 ng/mL at 9.33 h in rats, suggesting a similar distribution pattern in both rodents. However, the peak plasma concentrations and time (C_max_ and T_max_) of compound 4 were 197.35 mg/mL at 6 h in mice and 133 mg/mL at 10.67 h in rats, suggesting that the compound could be absorbed and distributed more efficiently in mice. Moreover, the peak plasma concentrations of compound 4 were higher than compound 3 in both animal species. The half-life (t_1/2)_ of compounds 3 and 4 in mice were 4.16 and 3.42 h, respectively, whereas the half-life in rats were 7.92 and 16.65 h, respectively. The results suggested that the compounds were maintained in the mice circulation for a shorter period compared to rats. The area under curve (AUC) of compound 3 were similar across species with AUC of 909.14 in mice and 1088.6 in rats; whereas, the AUC of compound 4 were different, 4175.74 in rats and 2745.4 in mice. The plasma concentrations of compound 3 in mice and rats, as well as the concentrations of compound 4 in mice, fell below the previously determined EC_90_ and EC_50_ (Figure 1(b)) within 24–48 h (Figure 5(c)). However, the plasma concentrations of compound 4 in rats at 48 h were still above the EC_90_ and EC_50_ levels suggesting incomplete clearance. Additionally, the toxicity of compounds in rats was evaluated by analyzing ALT and Cr levels from the plasma samples collected at 24, 48, and 72 h after compounds administration. Results were within normal limits and showed no significant difference between groups. Therefore, we concluded that the incomplete clearance of compound 4 in rats did not relate to any acute hepatorenal toxicity.

**Table 1.** The pharmacokinetic parameters of compounds 3 and 4 orally administered to mice and rats at 250 mg/kg.

	HL	Tmax	Cmax	AUClast	AUCINF_obs	AUC_% Extrap_obs	Vz_F_obs	Cl_F_obs	MRTlast	MRTINF_obs
(mean ± SD)	(h)	(h)	(mg/L)	(h*mg/L)	(h*mg/L)	(%)	(L/kg)	(L/h/kg)	(h)	(h)
3										
mice	4.163 ± 0.941	10.000 ± 3.464	68.9 ± 64.134	909.136 ± 834.595	909.37 ± 834.545	0.124 ± 0.185	7.963 ± 11.841	1.097 ± 1.534	10.545 ± 0.646	10.599 ± 0.728
rat	7.922 ± 6.558	9.333 ± 2.309	80.69 ± 39.89	1088.584 ± 646.006	1096.608 ± 632.618	2.128 ± 3.659	5.672 ± 8.054	0.339 ± 0.289	12.873 ± 5.726	14.532 ± 5.726
4										
mice	3.422 ± 0.419	6.000 ± 0.000	197.347 ± 77.044	2745.418 ± 927.253	2745.909 ± 927.381	0.018 ± 0.014	0.494 ± 0.200	0.100 ± 0.041	10.075 ± 0.356	10.085 ± 0.366
rat	16.649 ± 1.601	10.667 ± 2.309	133.303 ± 40.844	4175.743 ± 1640.975	4441.493 ± 1731.197	6.119 ± 0.999	1.596 ± 0.946	0.065 ± 0.032	22.233 ± 0.801	26.767 ± 0.127

Note: Abbreviations are elaborated in Supplementary Table S2.

### In vivo efficacy of compounds 3 and 4 against acute CHIKV infection

**Figure 6.**
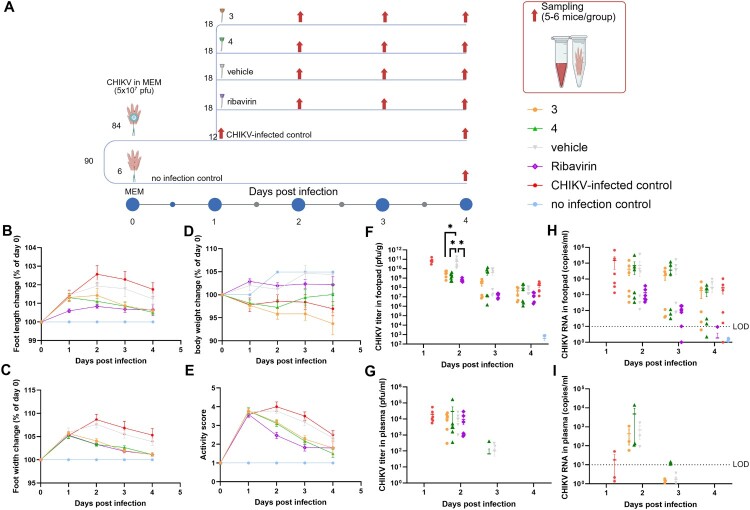
*In vivo* efficacy assessment of compounds 3 and 4 in a CHIKV-induced arthritis mouse model. (a) Schematic of the experimental design. C57BL/6 mice (n = 18/group) were infected with CHIKV (5 × 10^7^ PFU) via footpad injection and treated once daily with compound 3, compound 4 (250 mg/kg), ribavirin (50 mg/kg B.I.D), vehicle, or left untreated. An uninfected group served as a negative control (n = 6). Sampling of 6 mice/group was performed on days 1–4 post-infection. (b, c) Changes in foot length and width relative to day 0 (%). (d) Changes in body weight relative to day 0 (%). (e) Activity scores measured daily post-infection. (f) CHIKV titres in footpad tissues determined by plaque assay. (g) CHIKV titres in plasma determined by plaque assay. (h, i) CHIKV RNA levels in footpad tissues (h) and plasma (i) determined by RT-qPCR. Dashed line represents limit of detection (LOD). Each dot represents an individual mouse; horizontal lines indicate group means and standard deviation. Statistical significance was assessed using multiple paired t-test, Mann-Whitney test *p < 0.05.

A CHIKV-induced inflammation model in immunocompetent mice was used in this study as previously described (10) (Figure 6(a)). To evaluate the therapeutic potential of compounds 3 and 4 *in vivo*, immunocompetent C57BL/6 mice were inoculated with CHIKV and treated with a single oral dose of 250 mg/kg of either compounds (Figure 6(a)). Ribavirin (50 mg/kg, BID dose) and vehicle were included as controls, along with untreated CHIKV-infected and uninfected groups. Clinical parameters including foot length (Figure 6(b, c)), body weight (Figure 6(d)), and activity scores (Figure 6(e)) were monitored daily. On day 1 post-infection, CHIKV-infected mice developed notable foot swelling and elevated activity scores relative to non-infected controls (Figure 6(b)). Compound-treated groups exhibited moderate improvement in foot swelling and activity compared to the vehicle group, but these differences were transient and not significantly different from the vehicle control. Compound 3-treated mice also showed greater body weight loss than other groups (Figure 6(d)), possibly due to off-target effects on metabolism or absorption.

Viral titres were quantified by plaque assay (Figure 6(f, g)) and RT-qPCR (Figure 6(h, i)). Notably, both compounds significantly reduced CHIKV titres in footpad tissues at day 2 post-infection (Figure 6(f)), although there was no marked reduction in systemic viremia (Figure 6(g)). Viral RNA levels in footpad tissue also decreased over time in treated animals, with compound 3 showing a marked reduction by day 3 (Figure 6(h)). The reduction in viral RNA levels was more evident in tissue than in plasma, possibly due to delayed compound distribution and high inoculum levels interfering with detection. Plaque titration of footpad tissue revealed that both compounds 3 and 4 significantly reduced infectious viral titres on day 2, with compound 3 maintaining efficacy through day 3 howbeit not significantly (Figure 6(f)). However, RT-qPCR analysis of viral RNA in footpads and plasma did not show clear differences between treatment groups, suggesting that plaque assay – by detecting infectious virus – offers superior resolution for assessing antiviral activity. Viral RNA and titres in plasma remained low across all groups, indicating that infection was largely restricted to local tissue and did not result in systemic dissemination.

**Figure 7.**
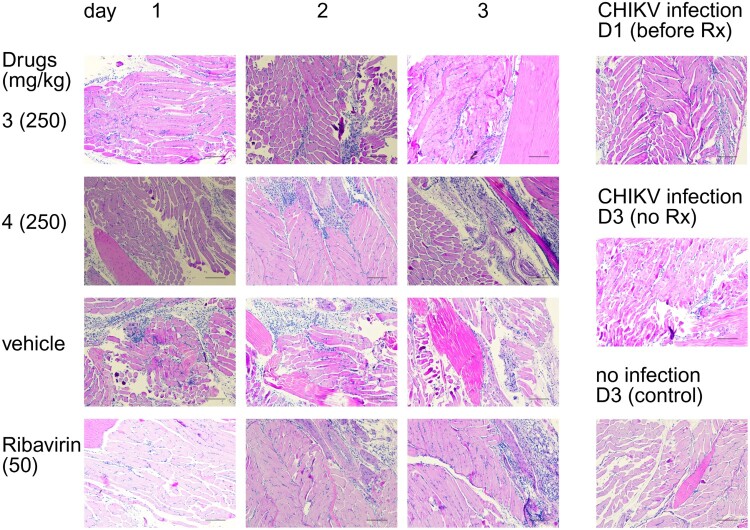
H & E pathology of footpad samples on day 1, 2, and 3 after administering a single dose of 250 mg/kg compounds 3 or 4. Ribavirin was used as a positive control (50 mg/kg, BID, IP, 6 doses). The line represents a 100 µm scale. Representative H&E-stained sections of footpad tissue collected on days 1–3 post-infection. Treatment groups include compound 3 (250 mg/kg), compound 4 (250 mg/kg), ribavirin (50 mg/kg), and vehicle. Additional controls include CHIKV-infected mice before treatment (day 1), CHIKV-infected untreated mice (day 3), and uninfected mice (day 3). Tissue sections were examined for inflammatory cell infiltration and structural changes.

Histological evaluation of footpad sections stained with hematoxylin and eosin (H&E) further supported the virological findings (Figure 7). Vehicle-treated mice exhibited marked polymorphonuclear (PMN) cell infiltration, edema, and tissue disruption – hallmarks of acute viral inflammation. In contrast, mice treated with compounds 3, 4, or ribavirin showed reduced PMN infiltration on day 1, indicating early modulation of local inflammatory responses. However, by days 2 and 3, neither compound nor ribavirin completely resolved inflammation, with PMN infiltration persisting at moderate levels. These findings are consistent with plaque assay results, which showed partial but not complete suppression of viral replication in the footpad tissue over time. Notably, the histological profile of ribavirin-treated mice closely resembled those of compounds 3 and 4, suggesting that the biscoumarins had comparable early anti-inflammatory and antiviral effects in this model.

Compounds 3 and 4 demonstrated early but transient antiviral and anti-inflammatory effects against CHIKV in a mouse arthritis model. Reduction in local viral titres and PMN infiltration was most apparent on day 1, though neither compound fully suppressed inflammation or viral replication through later stages. These results highlight partial efficacy and support further optimization of dosing, formulation, or combination strategies to enhance therapeutic potential.

## Discussion

This study demonstrates the antiviral potential of biscoumarin derivatives, particularly compound 3, against CHIKV in both *in vitro* and *in vivo* models. Compound 3, 3,3′-((3-chlorophenyl)methylene)bis(4-hydroxy-2H-chromen-2-one), exhibited potent antiviral activity in cell-based assays with EC₅₀ values of 2.85 ± 0.42 μM and 3.08 ± 0.45 μM in Vero and HEK293 cells, respectively, and selectivity indices >20. Its antiviral effect persisted when added as late as 12 hours post-infection (TOA assay). Additionally, a statistic analysis suggested the 3 and 4 – treated TOA were significantly different from their DMSO controls at early time-points (supplementary Figure S2.2), suggesting interference at the RNA replication stage of the CHIKV life cycle. However, viral replication resumed upon compound removal at 12 hpi (TOR assay), indicating a reversible and possibly non-cytotoxic mechanism of action. Consistent with these findings, compound 3 also inhibited a stably-expressed CHIKV replicon cell line with an IC₅₀ of 6.64 ± 0.86 μM, reinforcing its role in targeting intracellular stages such as viral RNA replication and/or protein translation. A similar pattern of inhibition was observed with compound 4, implying a shared or closely related mode of action.

Mechanistically, molecular docking and biochemical assays identified CHIKV nsP1 methyltransferase (MTase) as a potential viral target. Compounds 3 and 4 were predicted to bind at the SAM-binding site of nsP1 and exhibited moderate inhibition of MTase activity, although less potently than sinefungin. These findings suggest nsP1 as one of the molecular targets of biscoumarins, but also highlight that other cellular targets may contribute to the observed antiviral activity. Bioinformatic target prediction identified possible host targets such as PLK1, CASP6, and USP1 – proteins involved in cell cycle regulation and apoptosis, which are frequently exploited by viruses. Further studies, including siRNA knockdowns and binding validation via SPR, are needed to confirm these targets and elucidate the mechanism of inhibition. A structure–activity relationship (SAR) study will also be essential for optimizing compound efficacy and specificity.

In comparison to previous coumarin-based CHIKV inhibitors, biscoumarins demonstrated enhanced potency [39]. Earlier studies reported EC₅₀ values of 10.2–19.1 μM for uracil-coumarin-aromatic derivatives [40], 9.9–13.9 μM for coumarin-thioguanosine conjugates[9], and 0.5–10.7 μg/mL for natural coumarins from M. americana seeds [41]. Most of these compounds were proposed to act post-entry, aligning with the intracellular activity observed in this study. Therefore, it was likely that most coumarin derivatives inhibited the CHIKV replication by interfering with the viral activities inside the cells. A previous study showed a series of quinazoline-coumarin conjugates for their antiviaral activity against CHIKV. These compounds containing 2 core components – a quinazolinamine and coumarin moieties joined by a thiomethylene (–SCH_2_–) linker showed great inhibition against CHIKV with EC_50_ values ranging from 1.96 to 193 μM [10]. Unlike these structures, compounds 3 and 4 possess a rigid bis-coumarin scaffold and hydroxyl groups that may facilitate hydrogen bonding, while the chlorine substituent exerts electron-withdrawing effects, potentially enhancing target binding. This structural framework may provide higher specificity and reduced off-target effects compared to more flexible or substituted analogs.

Importantly, both compounds demonstrated favourable toxicity profiles *in vivo*. A single high oral dose (250 mg/kg) of either compound in mice and rats resulted in no significant signs of acute hepatorenal toxicity over 3–7 days of monitoring. Serum ALT and creatinine levels remained within normal physiological limits (Figure 5(e)), and activity scores were unaffected (Figure 4). Furthermore, no histopathological abnormalities were detected in liver or kidney tissues, supporting a lack of acute organ toxicity. These findings are notable given historical concerns over coumarin hepatotoxicity in rodents. The low toxicity and stability in aqueous solutions offer advantages for pharmacokinetic studies of compounds [42]. The low toxicity observed in this study suggests that the biscoumarin scaffold, particularly compound 3, may offer a safer chemical backbone for antiviral development, although chronic toxicity, genotoxicity, and off-target effects must be evaluated in future studies. However, multiple doses cannot be administered as the acute drug-induced hepatotoxicity and proximal renal tubular necrosis were observed (Supplementary Figure S6). Therefore, the safe dose was determined to 250 mg/kg, single dose.

In mice, the single high dose (250 mg/kg) of 3 and 4 did not show any acute hepatorenal toxicity within 7 days of administration, and there was no significant change in activity scores (Figure 4). Similar findings were obtained in rats where both ALT and Cr levels were within normal limits (Figure 5(e)) within 3 days. The pharmacokinetics suggested that a single administration could be sufficient to treat the CHIKV infection as the drug concentrations were retained above the EC_50_ and EC_90_ levels for at least 12 h (Figure 5(c, d)). Previously, the toxicity and pharmacokinetics of biscoumarin have not been explored, but those of coumarin and its major metabolite, 7-hydroxycoumarin (7HC), have been extensively studied for decades. After absorption, the coumarin went through an extensive first-pass effect by cytochrome P450 2A6 called a 7-hydroxylation pathway and subsequently to glucuronide and sulphate conjugates of 7-hydroxycoumarin (7HC) [43]. Only 2–6% of coumarin reached the systemic circulation and was detectable in human plasma [44]. The rapid excretion of coumarin and its metabolites occurs through urine. On the contrary to the case of humans, coumarin was more toxic to rodents as they utilized a 3,4-epoxidation as their major drug metabolism pathway. The coumarin 3,4-epoxide was readily converted to o-hydroxyphenylacetaldehyde (o-HPA) [45]. The o-HPA was a prominent coumarin metabolite in rat liver microsomal incubations, contributing to the hepatotoxicity in rats [45]. Moreover, a comparative kinetics study observed a five-times longer plasma half-life of compound in rats than in mice [46]. Similarly, our compounds 3 and 4 showed a ∼1.9 and ∼4.9-times longer plasma half-life in rats than in mice, respectively (Table 1). Overall, the compound 3 showed similar pharmacokinetic profiles in mice and rats whereas the compound 4 was more efficiently distributed and excreted in mice than rats.

A CHIKV-induced inflammation model in immunocompetent mice was used in this study. Mouse models used for studying acute CHIKV infection is broadly classified into 3 models – the neonatal mice model, immunocompromised mouse model and the arthritis/myositis model. While neonatal mice have underdeveloped immune systems and immunocompromised mice lack the type 1 interferon and could easily die, the arthritis model which is an immunocompetent mice model induces arthritis (a common CHIKV disease symptom) upon infection. For *in vivo* efficacy, the immunocompetent C57BL/6 CHIKV arthritis model was used, which closely mimics human infection through localized footpad inflammation and viral dissemination to musculoskeletal and lymphoid tissues. This model offers advantages over neonatal or immunocompromised systems by preserving type I interferon responses and allowing assessment of both viral replication and host inflammatory pathology. Applications of the immunocompetent model include pathogenesis, vaccines, and drug treatment studies [47,48].

In this setting, compound 3 significantly reduced footpad swelling, viral loads in infected tissues, and systemic viremia as confirmed by plaque assay in footpad tissue (Figure 6(c)). These effects underscore its ability to reduce both viral burden and inflammation *in vivo*. Interestingly, viral genome detection in plasma was limited, likely due to rapid clearance or high local inoculum in the footpad impeding systemic spread. A recent study emphasized the importance of early treatment initiation, as efficacy of antiviral agents dramatically decreases when administered 24 h post-infection [49]. Moreover, the limitation with only a single dose 250 mg/kg could attenuate the treatment option. Future work should investigate earlier time points, alternative dosing strategies, and optimizing the formulary of compound 3 for better absorption and distribution. Identification of targets of compounds would be important for the design of more effective compounds. However, caution is needed in modifying chemical structures as a single difference in positioning a Cl atom, present in compounds 3 and 4, affected the pharmacokinetics and antiviral efficacy profiles of compounds. Therefore, further investigation should emphasize on optimizing the formulary of compound 3 for better antiviral activity as well as absorption and distribution.

Taken together, our findings highlight compound 3 as a promising lead for anti-CHIKV therapy, with demonstrated intracellular antiviral activity, favourable pharmacokinetics, low toxicity, and *in vivo* efficacy. The rigid biscoumarin framework and electron-withdrawing substituents likely contribute to high target specificity and reduced off-target effects. However, caution must be exercised in chemical modification, as even minor structural changes – such as halogen placement – significantly affect pharmacokinetic and antiviral profiles. Going forward, emphasis should be placed on optimizing compound formulation for enhanced absorption and tissue distribution, conducting long-term safety assessments, and validating viral and host targets through biochemical and cellular approaches. These efforts will inform the rational design of more effective and selective biscoumarin-based antivirals.

## Supplementary Material

Supplementary_TP_CHIKV_R2.docx
