# Supplementary material for: Chlorinated biscoumarins inhibit chikungunya virus replication in cell-based and animal models
Source: Emerg Microbes Infect. 2025 Jul 3;14(1):2529889. doi: 10.1080/22221751.2025.2529889 (PMC12305873; doi:10.1080/22221751.2025.2529889)
Supplement: Supplementary_TP_CHIKV_R2.docx [file TEMI_A_2529889_SM5435.docx]

Supplementary Figures and Tables

Supplementary Figure S1: Validation parameters in pharmacokinetic analysis

Supplementary Fig. S1.1-1.2 LC-MS/MS spectra of compounds 3 (445.050→161.100) and 4 (445.050→161.500)

Y = (17172.4)X + (-174.641)

R^2^= 0.9984629

Supplementary Fig. S1.3 Calibration curve of compound 3 at 0.0781- 20.000 µg/ml

Y = (9345.45)X + (-172.133)

R^2^= 0.9996684

Supplementary Fig S1.4 Calibration curve of compound 4 at 0.098-50.000 µg/ml

Supplementary Table S1.1 Calibration curve and back-calculated concentrations for TP074 in blank rat plasma

| **Parameter** | **Back-calculated concentrations of TP074 in blank rat plasma**  **(µg /ml)** | | | | | | | | **Equation of standard curve** | **R^2^** |
| --- | --- | --- | --- | --- | --- | --- | --- | --- | --- | --- |
|  | **CC1** | **CC2** | **CC3** | **CC4** | **CC5** | **CC6** | **CC7** | **CC8** | Y = (9345.45)X + (-172.133) | 0.99967 |
| Back cal. | 0.109 | 0.351 | 1.558 | 3.262 | 6.039 | 12.206 | 24.883 | 50.518 |  |  |
| % Accuracy | 111.224 | 89.770 | 99.680 | 104.384 | 96.624 | 97.648 | 99.532 | 101.036 |  |  |
| Nominal value (µg /ml) | 0.098 | 0.391 | 1.563 | 3.125 | 6.250 | 12.500 | 25.000 | 50.000 |  |  |

Supplementary Table S1.2 Calibration curve and back-calculated concentrations for TP103 in blank rat plasma.

| **Parameter** | **Back-calculated concentrations of TP103 in blank rat plasma (µg/ml)** | | | | | | | | **Equation of standard curve** | **R^2^** |
| --- | --- | --- | --- | --- | --- | --- | --- | --- | --- | --- |
|  | **CC1** | **CC2** | **CC3** | **CC4** | **CC5** | **CC6** | **CC7** | **CC8** |  |  |
| Back cal. | 0.078 | 0.299 | 0.656 | 1.259 | 2.546 | 5.028 | 10.208 | 18.771 | Y = (17172.4)X + (-174.641) | R^2^= 0.9984629 |
| % Accuracy | 100.378 | 95.568 | 105.021 | 100.685 | 101.859 | 100.553 | 102.083 | 93.853 |  |  |
| Nominal value (µg/ml) | 0.0781 | 0.3125 | 0.625 | 1.250 | 2.500 | 5.000 | 10.000 | 20.000 |  |  |

Supplementary Table S2: Abbreviations of pharmacokinetics in Table 2

| Abbreviation | Full Term | Definition |
| --- | --- | --- |
| HL | Half-life | The time required for the concentration of the drug in the plasma to decrease by half. |
| Tmax | Time to Maximum Concentration | The time after drug administration at which the maximum plasma concentration (Cmax) is observed. |
| Cmax | Maximum Concentration | The highest observed plasma concentration of a drug after administration. |
| AUClast | Area Under the Curve to Last Time Point | The area under the plasma concentration–time curve from time zero to the last measurable concentration. |
| AUCINF_obs | Area Under the Curve to Infinity (Observed) | The area under the plasma concentration–time curve from time zero extrapolated to infinity, using the observed data. |
| AUC_% Extrap_obs | Percent Extrapolated AUC (Observed) | The percentage of the total AUC (to infinity) that is due to extrapolation beyond the last measured time point. |
| Vz_F_obs | Apparent Volume of Distribution (Observed) | The volume in which the drug would need to be uniformly distributed to give the observed plasma concentration, based on observed data and oral dosing. |
| Cl_F_obs | Apparent Clearance (Observed) | The volume of plasma from which the drug is completely removed per unit time, based on observed data and oral dosing. |
| MRTlast | Mean Residence Time to Last Time Point | The average time a drug molecule remains in the body up to the last measured concentration. |
| MRTINF_obs | Mean Residence Time to Infinity (Observed) | The average time a drug molecule remains in the body, based on the observed data extrapolated to infinity. |


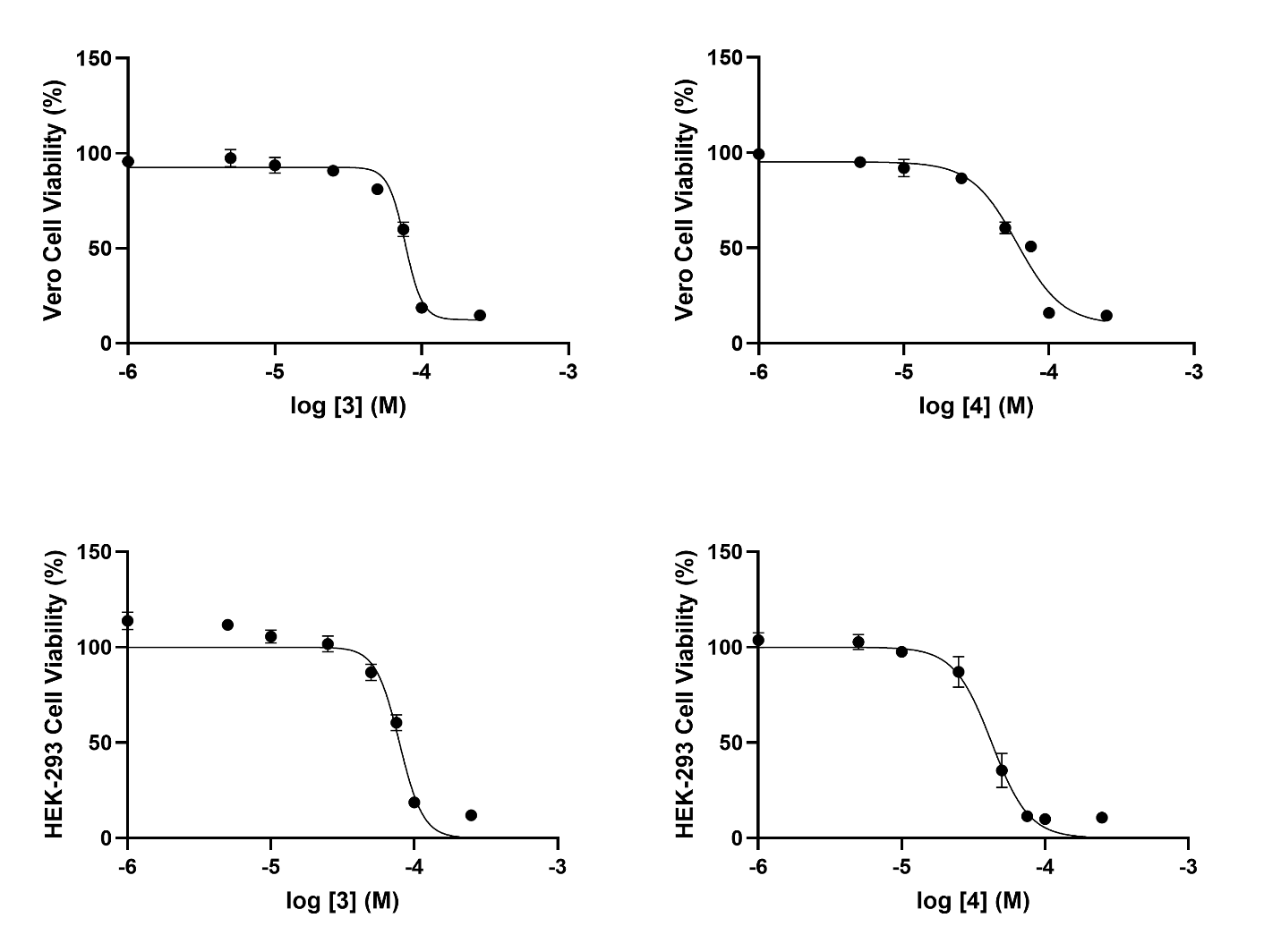


Supplementary Figure S2.1 The graphs represented CC50s of 3 and 4 in Vero and HEK-293 cells. The CC50s of both compounds in Vero were 75.85±3.25 and 66.97±4.86 µM, respectively and the CC50s in HEK293 were 71.60±3.67 and 44.35±1.48, respectively. Results were means and standard deviations of three independent experiments.


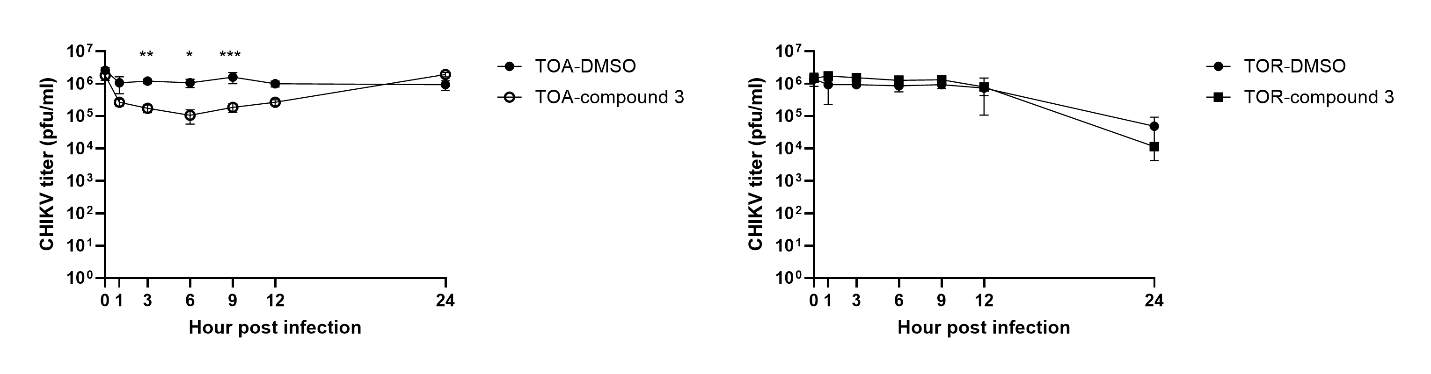


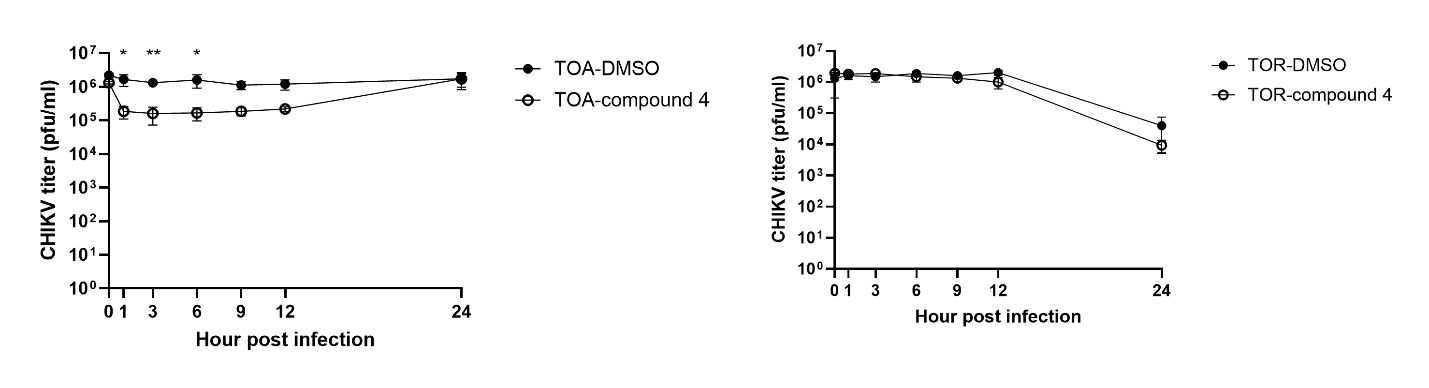
 Supplementary Figure S2.2 The 2-way ANOVA with Bonferroni’s multiple comparison tests of TOA and TOR of the compounds 3 and 4 with their respective DMSO controls.

**Supplementary 3. Bioinformatics-based target analysis**

To explore possible mechanisms underlying the antiviral effects of compounds 3 and 4, we performed a bioinformatics-based target prediction using the Chemical Similarity Ensemble Approach (SEA). Structurally similar compounds (≥95% similarity based on Tanimoto scores) were identified, and their bioactivity profiles were retrieved from PubChem. Thirteen compounds shared notable structural similarity and were associated with 29 protein targets, visualized using a Cytoscape interaction network. Multidimensional scaling (MDS) clustering grouped these compounds based on structural similarity, revealing four distinct clusters. Only the main cluster, which included compounds 3 and 4, was considered for further analysis. Although this approach is exploratory, it provides preliminary insight into potential targets that may contribute to the antiviral effect.

Among the predicted targets, polo-like kinase 1 (PLK1) has been previously reported to interact with CHIKV infection pathways. Caspase 6 (CASP6) and ubiquitin-specific peptidase 1 (USP1), which are involved in apoptosis and ubiquitin signaling respectively, may also play a role during infection. Other targets, such as ADAM17, PGE2 receptor, and ALOX12, are associated with inflammation and bone damage. While these predictions require experimental validation, they may help guide future studies on potential host-based adjunctive targets modulated by biscoumarin derivatives.


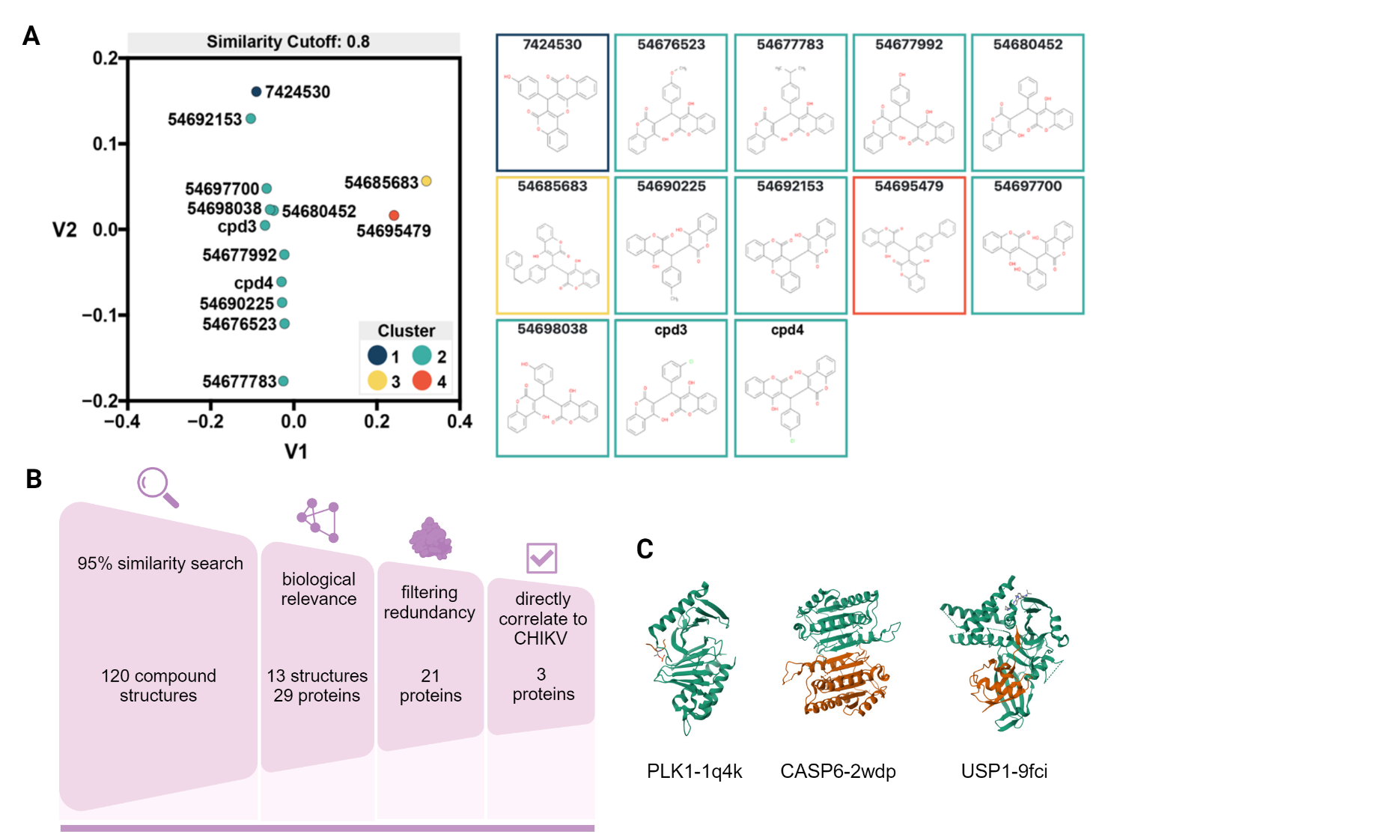


**Supplementary Figure S3** **Bioinformatic-based target prediction** A) Multidimensional Scaling (MDS) clustering of 13 similar compounds, including potent compounds 3 and 4, based on a similarity cutoff of 0.8, is presented as an XY scatter plot. The scatter plot displays the compound IDs (cids) and their corresponding 3D structures, with data points colored according to their respective clusters. B) the schematic of bioinformatic-related search and refinement C) three predicted targets and their RSCB IDs.

**Supplementary Table S3** List of compounds ID (CID) with their reported targets and the correlation to CHIKV infection and chronic inflammation

| cid | Cluster | Target name | Correlate to CHIKV infection |
| --- | --- | --- | --- |
| 54677783 | 1 | ADAM10 - ADAM metallopeptidase domain 10 (human) | - |
| 54677783 | 1 | ADAM17 - ADAM metallopeptidase domain 17 (human) | indirect |
| 54677783 | 1 | ALOX12 - arachidonate 12-lipoxygenase, 12S type (human) | inflammation |
| 54677783 | 1 | CASP6 - caspase 6 (human) | direct |
| 54677783 | 1 | CRHBP - corticotropin releasing hormone binding protein (human) | - |
| 54677783 | 1 | CRHR2 - corticotropin releasing hormone receptor 2 (human) | - |
| 54677783 | 1 | CYP2C9 - cytochrome P450 family 2 subfamily C member 9 (human) | - |
| 54677783 | 1 | NQO1 - NAD(P)H quinone dehydrogenase 1 (human) | indirect |
| 54677783 | 1 | NR2E3 - nuclear receptor subfamily 2 group E member 3 (human) | - |
| 54677783 | 1 | PLK1 - polo like kinase 1 (human) | direct |
| 54677783 | 1 | POLH - DNA polymerase eta (human) |  |
| 54677783 | 1 | POLK - DNA polymerase kappa (human) |  |
| 54677783 | 1 | PTGER2 - prostaglandin E receptor 2 (human) | inflammation |
| 54677783 | 1 | RAPGEF3 - Rap guanine nucleotide exchange factor 3 (human) | - |
| 54677783 | 1 | RAPGEF4 - Rap guanine nucleotide exchange factor 4 (human) | - |
| 54677783 | 1 | RdRp - putative polyprotein (Crimean-Congo hemorrhagic fever orthonairovirus) | - |
| 54677783 | 1 | USP1 - ubiquitin specific peptidase 1 (human) | direct |
| 54677992 | 1 | NQO1 - NAD(P)H quinone dehydrogenase 1 (human) | indirect |
| 54676523 | 1 | NQO1 - NAD(P)H quinone dehydrogenase 1 (human) | indirect |
| 54692153 | 1 | NQO1 - NAD(P)H quinone dehydrogenase 1 (human) | indirect |
| 54680452 | 1 | NQO1 - NAD(P)H quinone dehydrogenase 1 (human) | indirect |
| 54680452 | 1 | AICDA - activation induced cytidine deaminase (human) | - |
| 54698038 | 1 | AICDA - activation induced cytidine deaminase (human) | - |
| 54690225 | 1 | ALOX12 - arachidonate 12-lipoxygenase, 12S type (human) | inflammation |
| 54697700 | 1 | Impa1 - inositol monophosphatase 1 (Norway rat) | - |
| 7424530 | 2 | HTT - huntingtin (human) | indirect |
| 54685683 | 3 | Integrase (Human immunodeficiency virus 1) | - |
| 54685683 | 3 | NQO1 - NAD(P)H quinone dehydrogenase 1 (human) | indirect |
| 54695479 | 4 | NQO1 - NAD(P)H quinone dehydrogenase 1 (human) | indirect |
|  |  |  |  |


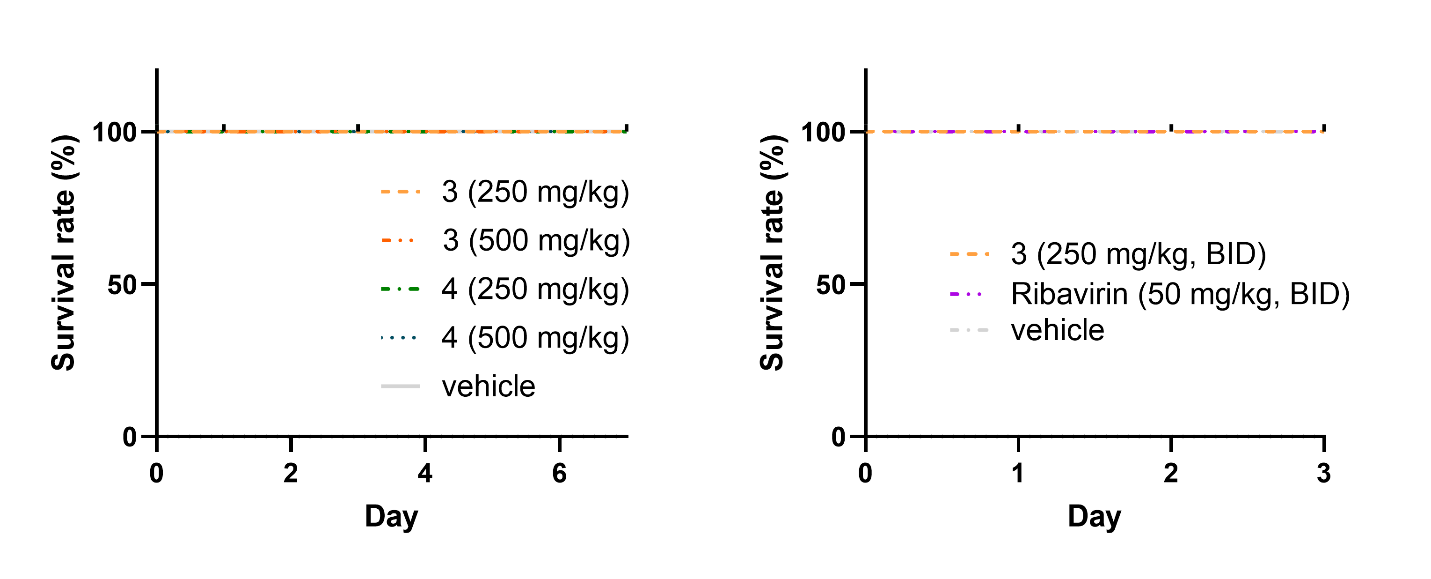


Supplementary Figure S4 Survival analysis of animals in acute toxicity experiment


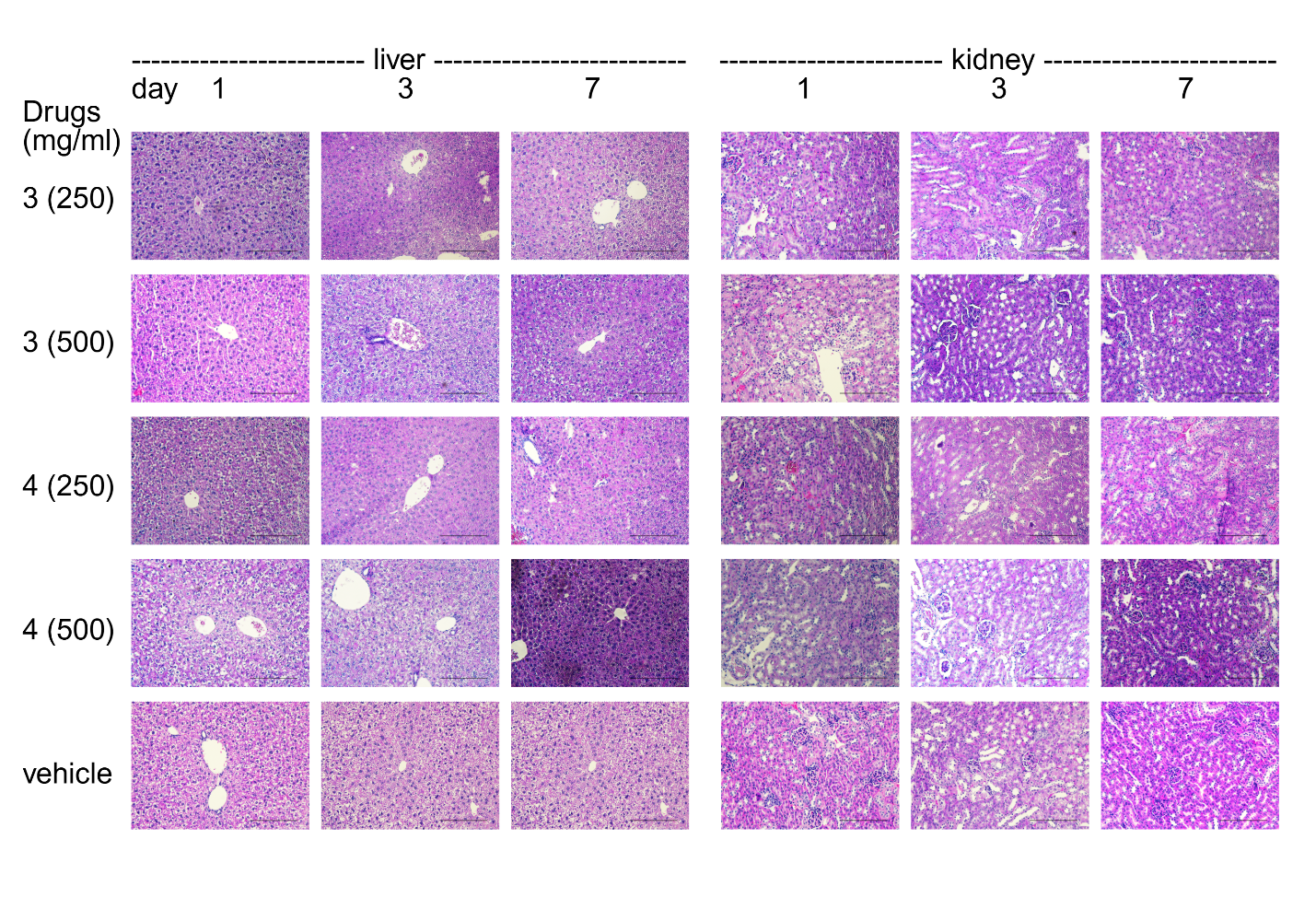


Supplementary Figure 5 H & E pathology of liver and kidney samples on day 1, 3, and 7 after administering a single dose of 250 and 500 mg/kg compounds 3 or 4. The line represents a 100 µm scale.


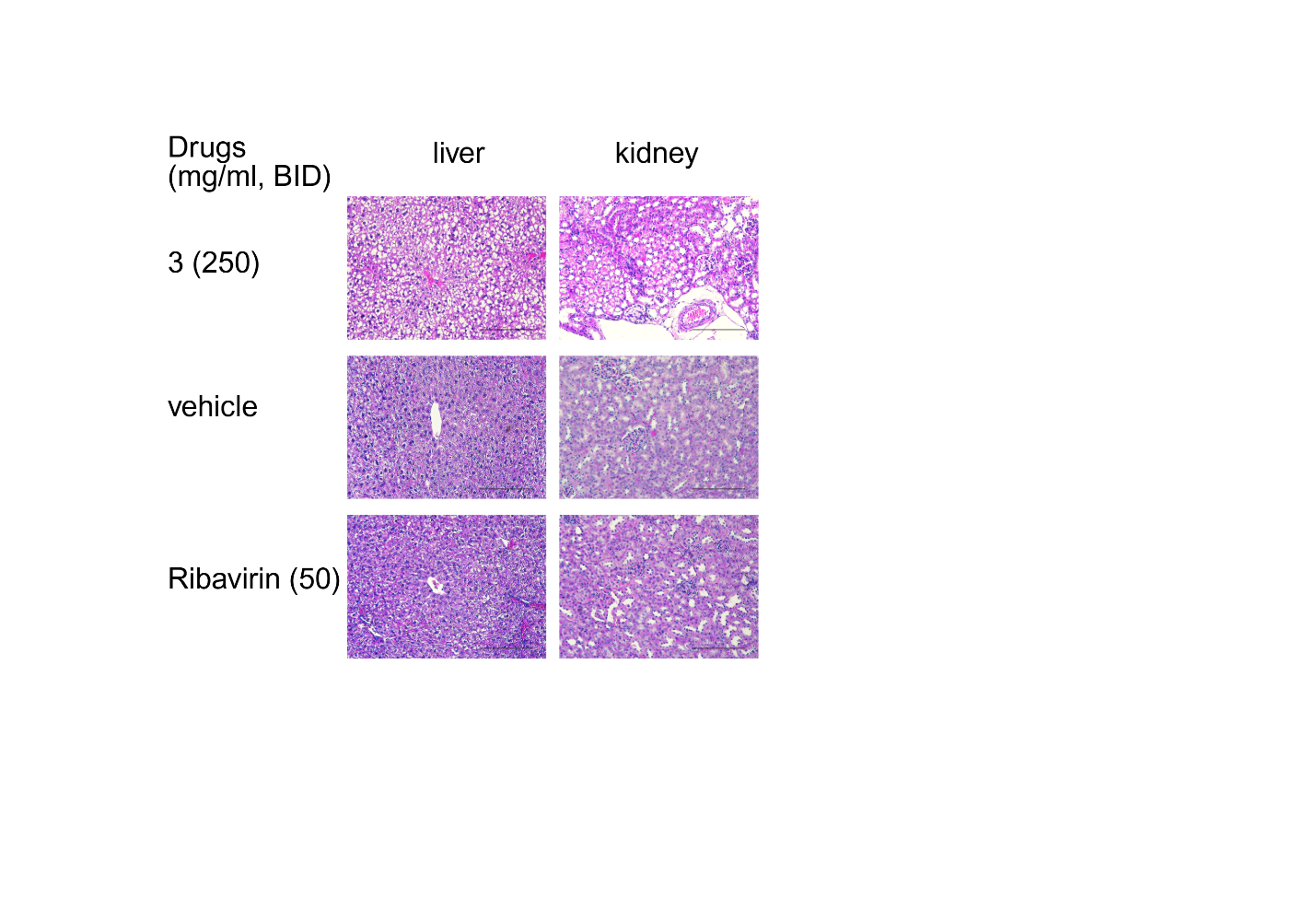


Supplementary Figure 6 H & E pathology of liver and kidney samples on day 3 after administering multiple (6) doses of 250 mg/kg compounds 3. The line represents a 100 µm scale.


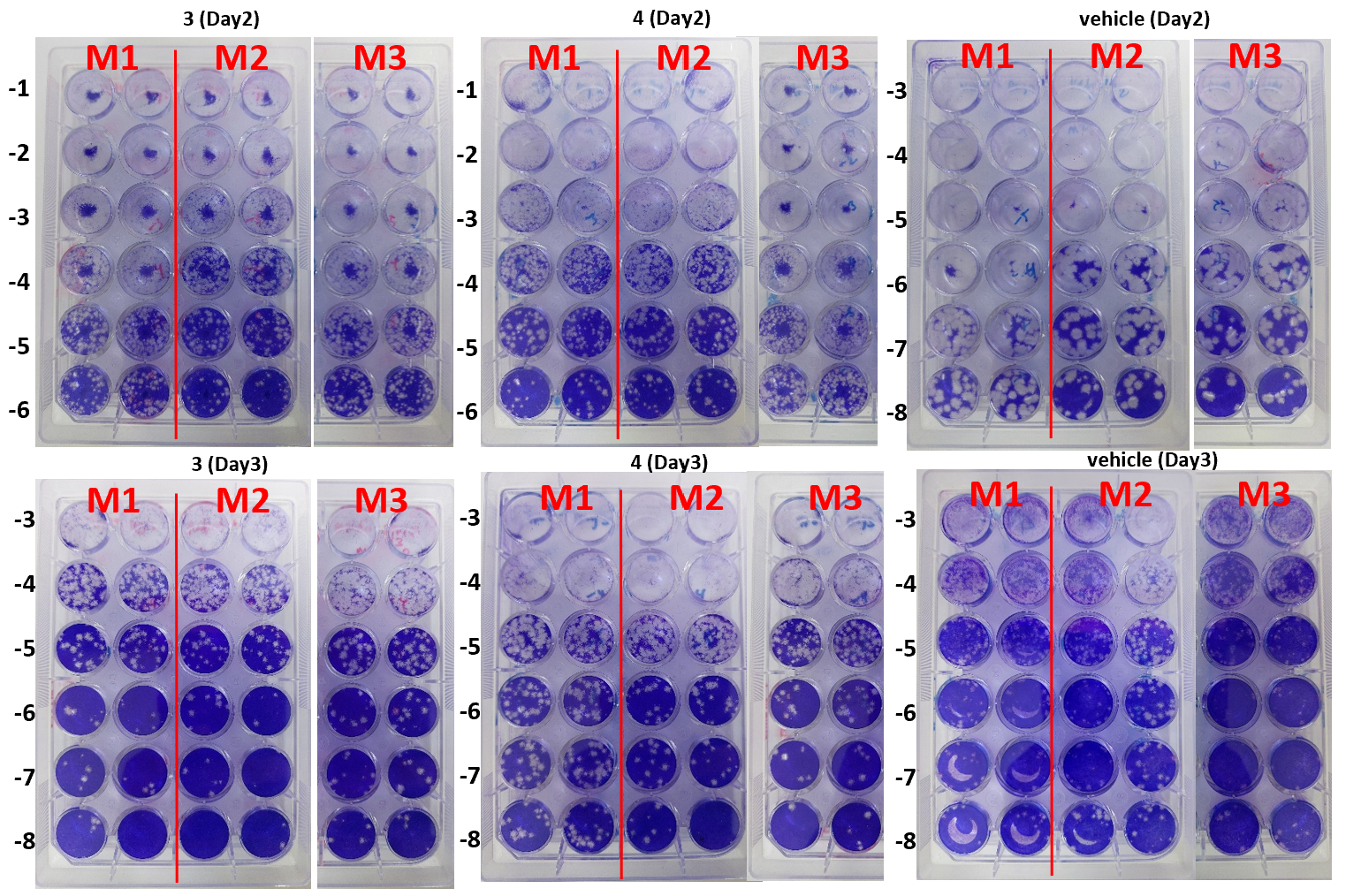

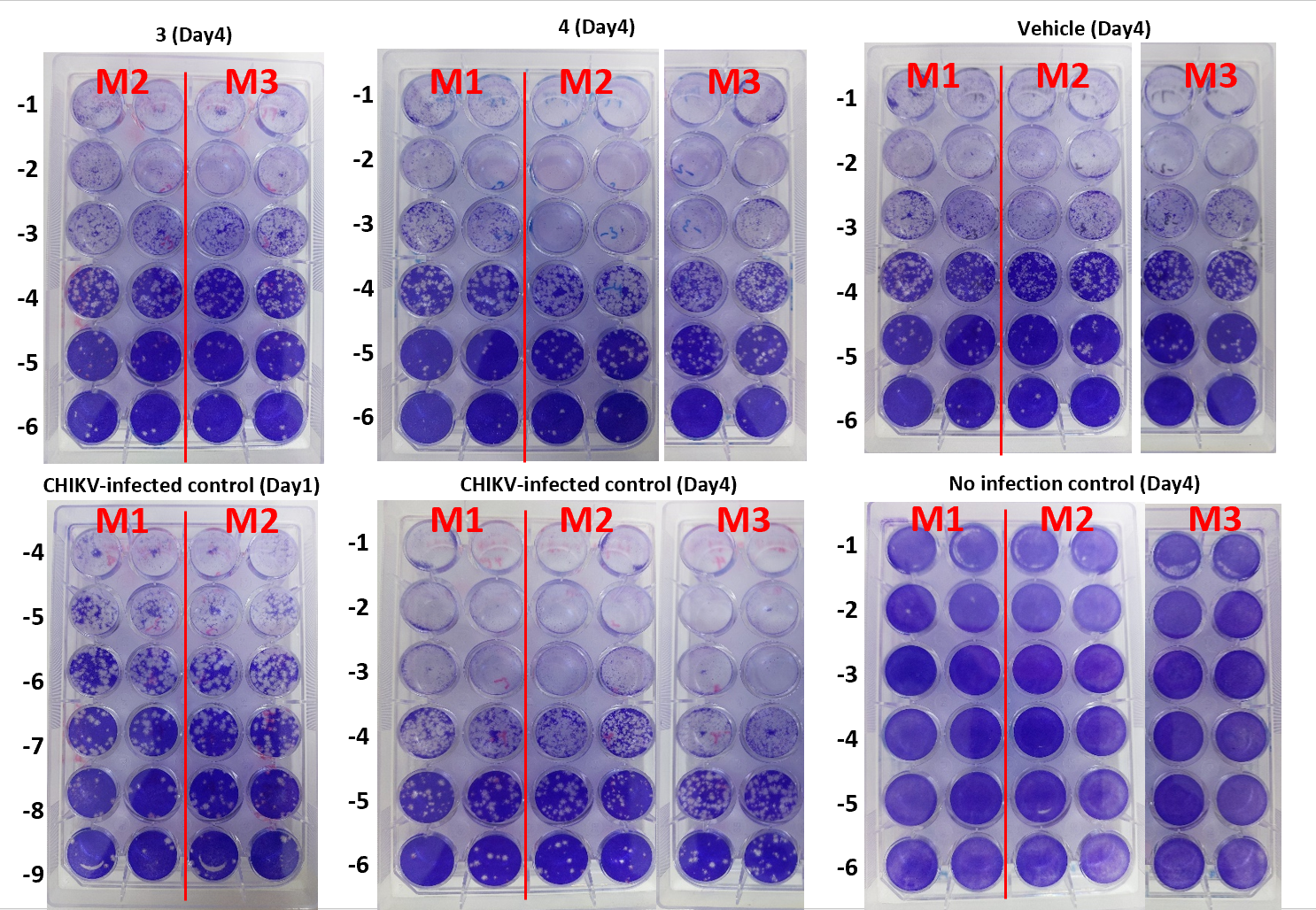


Supplementary Figure S7 Plaque assays of tissue viral loads in the in vivo efficacy test


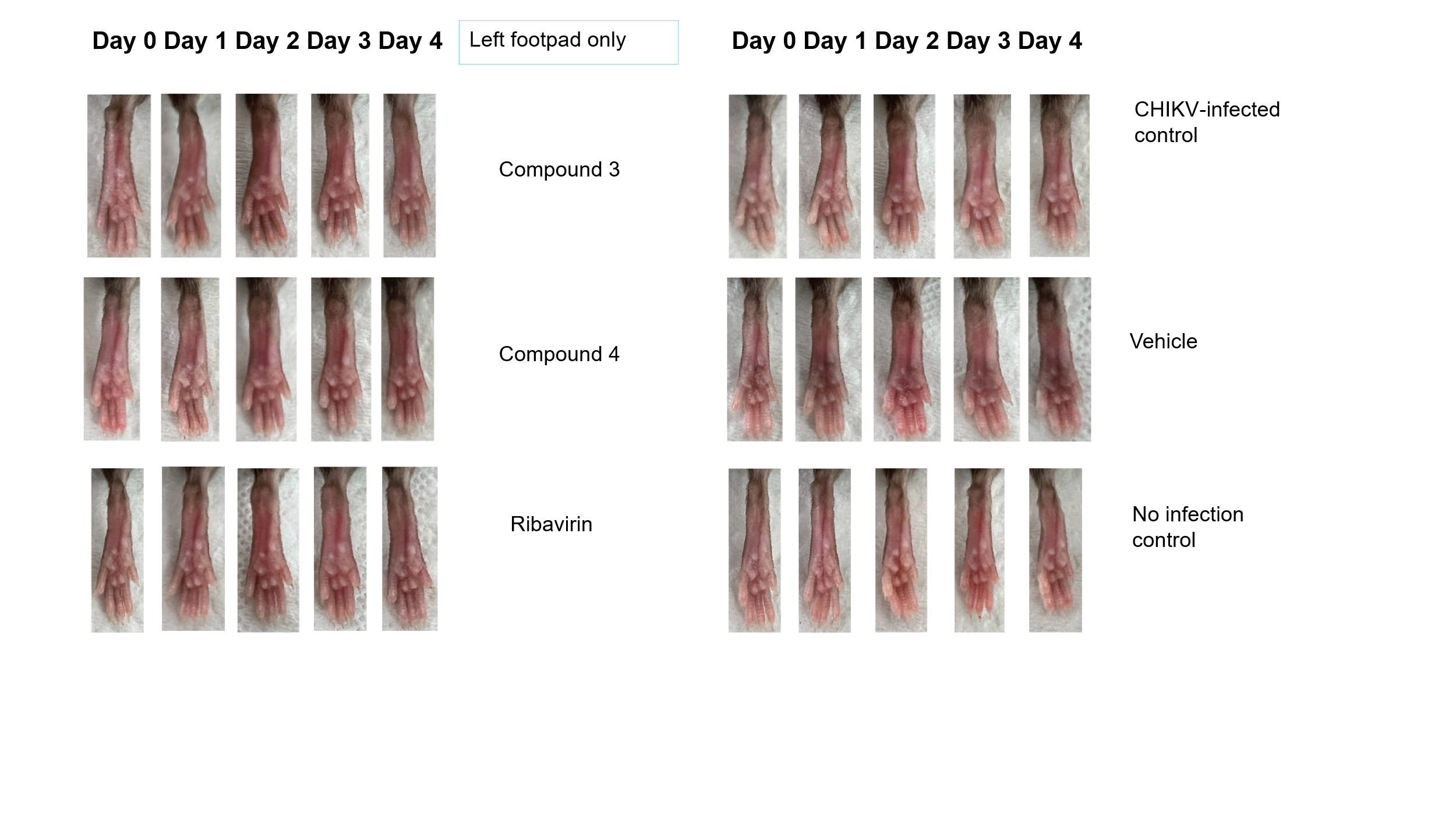
Supplementary Figure S8 Gross anatomy of Lt footpad in the in vivo efficacy test
